# Silencing MALAT1 represses pathological progression, inflammation, and vascular smooth muscle cell phenotype switching by regulating the SEMA3C-mediated Smad pathway in intracranial aneurysms

**DOI:** 10.3389/fncel.2026.1706518

**Published:** 2026-03-11

**Authors:** Junlong Kang, Wei Li, Xinjie Gao, Xinhua Tian, Wei Feng, Xiang Yao, Feng Wei, Luyue Chen, Hongjin Chen, Junjiang Tong, E Chen, Yuxiang Gu

**Affiliations:** 1 Department of Neurosurgery, The First Affiliated Hospital of Fujian Medical University, Fuzhou, China; 2 Department of Neurosurgery, Zhongshan Hospital of Xiamen University, School of Medicine, Xiamen University, Xiamen, China; 3 Department of Pharmacy, The Third Hospital of Xiamen, Xiamen, China; 4 Department of Neurosurgery, Huashan Hospital of Fudan University, Shanghai, China; 5 Neurosurgical Institute of Fudan University, Shanghai, China

**Keywords:** intracranial aneurysm, MALAT1, semaphorin 3C, smadpathway, vascular smooth muscle cells

## Abstract

**Background:**

The crucial role of metastasis-associated lung adenocarcinoma transcript 1 (MALAT1) in regulating aneurysm formation, inflammation, and neural dysfunction has gradually been recognized. This study aimed to evaluate the effects of MALAT1 modification on pathological changes, inflammation, vascular smooth muscle cell (VSMC) phenotype switching, and the underlying mechanism in intracranial aneurysms (IAs).

**Methods:**

MALAT1-overexpressing (oeMALAT1), MALAT1 short hairpin (shMALAT1), and semaphorin 3C (SEMA3C)-overexpressing (oeSEMA3C) lentiviruses were transfected alone or in combination into basilar artery VSMCs originating from IA rats. These lentiviruses were then stereotactically injected into IA rats.

**Results:**

*In vitro*, the overexpression of MALAT1 inhibited cell proliferation while promoting cell apoptosis and invasion; the release of TNF-α, IL-1β, and IL-6; and the transformation of IA basilar artery VSMCs from the contractile type to the synthetic type. However, the silencing of MALAT1 had the opposite effect. The silencing of MALAT1 downregulated SEMA3C, and its silencing inactivated the Smad pathway. Furthermore, SEMA3C overexpression attenuated the effects of MALAT1 silencing on IA basilar artery VSMC proliferation, apoptosis, invasiveness, proinflammatory cytokines, phenotype switching, and Smad pathway inactivation. *In vivo*, silencing MALAT1 reduced the release of TNF-α, IL-1β, and IL-6, decreased pathological progression, inhibited VSMC synthetic type switching, and inactivated the Smad pathway in IA rats. However, the overexpression of SEMA3C reversed these effects.

**Conclusion:**

Silencing MALAT1 represses pathological progression, inflammation, and VSMC phenotype switching by regulating the SEMA3C-mediated Smad pathway in IA.

## Introduction

Intracranial aneurysm (IA) is a congenital structural abnormality or acquired pathological change in the artery wall and is one of the most common cerebrovascular diseases leading to disability or death (Ellis et al., 2018; Morel et al., 2022; Rahmani et al., 2022). IA frequently occurs, and its incidence can reach 3.2% (95% confidence interval: 1.9–5.2%) in the normal population without underlying disease (Vlak et al., 2011). Abnormalities in vascular smooth muscle cells (VSMCs), including the abnormal arrangement and apoptosis of VSMCs in intracranial artery vessel walls, play crucial roles in the pathogenesis of IA (Xu and Fang, 2023; Zhou et al., 2023). Hence, identifying potential targets that might participate in regulating the VSMC phenotype would be beneficial for better understanding the mechanism of IA occurrence and a potential way to restrain the progression of IA.

Metastasis-associated lung adenocarcinoma transcript 1 (MALAT1), a non-coding RNA, was first reported in lung cancer and is related to many biological processes *in vivo*, such as tumor metastasis, migration, and invasion (Li et al., 2024; Tasnim et al., 2024; Xia et al., 2025). Recently, the important role of MALAT1 in regulating hemangioma formation, inflammation, and neural cell dysfunction has been recognized (Dong et al., 2025; Gheidari et al., 2024; Rehman and Tamagnone, 2013; Shen et al., 2025; Wang et al., 2020; Xiao et al., 2017; Yan et al., 2020). For example, one study reported that MALAT1 is highly expressed in calcified leaflet tissue and that the overexpression of MALAT1 could promote the osteogenic differentiation of valve interstitial cells (VICs), the main cellular components of aortic valve leaflets (Xiao et al., 2017). In another study, the silencing of MALAT1 inhibited hemangioma growth (Wang et al., 2020). Furthermore, the knockdown of MALAT1 might reduce inflammation and release matrix metalloproteinases (MMPs) in VSMCs (Yu et al., 2022). Besides, MALAT1 also play important role in regulating the pathological process of the vascular disease, such as atherosclerosis and vascular calcification (Cremer et al., 2019; Gong et al., 2023). Therefore, it is reasonable to hypothesize that MALAT1 also plays an important role in regulating the cell phenotype of VSMCs, but related evidence is scarce.

Semaphorin 3C (SEMA3C), a family member of semaphorin proteins, is a secreted signaling protein in vertebrates that promotes axon growth in human dopaminergic neurons, guiding motor neurons to specific targets (Rehman and Tamagnone, 2013). SEMA3C also plays an important role in regulating the migration and phenotypic transition of VSMCs, as well as the development of the cardiovascular system (Lepore et al., 2006; Niimi et al., 2024; Plein et al., 2015). A recent study indicated that MALAT1 might be an upstream regulator of SEMA3C in cancer cell signal transduction (Wernig-Zorc et al., 2024). However, the relationship between MALAT1 and SEMA3C in the regulation of IA pathogenesis has not been reported.

Therefore, this study aimed to evaluate the effects of MALAT1 modification on regulating the VSMC phenotype, pathological changes, inflammation, and underlying mechanism to elucidate the role of MALAT1 in the pathogenesis and progression of IA.

## Materials and methods

### IA rat model establishment

IA rats (SLAC, China) were generated via methods described in previous studies with slight modifications (Hosaka et al., 2014; Li S. et al., 2020; Shi Y. et al., 2019). In brief, the left common carotid artery and right renal artery were ligated after the rats were anesthetized via inhalation of 3% pentobarbital sodium. The rats were stereotactically injected with 10 μL elastase (10 U/mL, MCE, China) at the right basal cistern 1 week after ligation. Then, osmotic pumps (ALZET, United States) containing angiotensin II (500 ng/kg/min, MCE, China) were implanted into subcutaneous pockets, which were made in the dorsal skin between the scapulae of the rats. The rats were fed for 4 weeks after osmotic pump implantation.

### VSMC isolation and culture

The basilar artery was harvested after the IA rat was constructed. VSMCs were isolated via methods described in a previous study (Cao et al., 2013). In brief, the interlayers of vessels were separated, cut into pieces and plated in a culture flask for 5–7 days. The VSMCs that migrated from the tissues were cultured in DMEM/F12 medium supplemented with 10% FBS (Procell, China) and passaged when the confluence reached 90%. VSMCs between 3 and 8 passages were used for further experiments.

### Lentivirus infection

MALAT1 overexpression (oeMALAT1) lentivirus (Genechem, China), negative control overexpression (oeNC) lentivirus (Genechem, China), MALAT1 short hairpin (shMALAT1) lentivirus (Genechem, China) and negative control short hairpin (shNC) lentivirus (Genechem, China) were used to infect the VSMCs at a multiplicity of infection (MOI) of 20:1. After infection, the VSMCs were classified into oeMALAT1, oeNC, shMALAT1 and shNC groups. VSMCs without infection were used as the control group.

Afterward, the shMALAT1 lentivirus, shNC lentivirus, SEMA3C overexpression (oeSEMA3C) lentivirus (Genechem, China), and oeNC lentivirus were used to coinfect the VSMCs at an MOI of 20:1. The VSMCs were divided into shNC + oeNC, shMALAT1 + oeNC, shNC + oeSEMA3C and shMALAT1 + oeSEMA3C groups. VSMCs without infection were used as the control group.

### Cell proliferation, apoptosis, and transwell assays

Cell proliferation was analyzed via a cell counting kit-8 (CCK-8; Servicebio, China). The VSMCs were plated in 96-well plates. At 0, 24, 48, and 72 h, the CCK-8 reagent was added to the wells, which were subsequently incubated for another 2 h. The absorbance at 450 nm was measured with a microplate reader (Biotek, United States). A TUNEL cell apoptosis detection kit (Servicebio, China) was used to analyze VSMC apoptosis at 72 h after infection. After being fixed with 4% paraformaldehyde, the VSMCs were incubated in TUNEL working solution and permeabilized with 0.3% Triton X-100. An inverted microscope (Motic, China) was used to capture images. The transwell assay was performed with a transwell insert (NEST, China). The VSMCs were seeded and cultured in transwell inserts for another 24 h at 72 h after infection. The invading VSMCs were imaged and counted after being stained with crystal violet (Servicebio, China). For TUNEL and transwell assays, five randomly selected fields were analyzed for each sample. Moreover, apoptotic cell rate was calculated using TUNEL-positive cell number divided by the total number of DAPI-stained cell nuclei.

### Western blot

The 72 h-infected VSMCs were lysed with RIPA buffer (Servicebio, China), which was added with protease and phosphatase inhibitor cocktails (Servicebio, China). The concentration of total protein was measured with a BCA kit (Servicebio, China). A total of 20 μg of thermally denatured protein was separated on a precast gel and transferred to a nitrocellulose membrane (Whatman, United States). BSA (5%) was used to block the membrane. Primary antibodies, including α-SMA (1:2,000, Servicebio, China), OPN (1:2,500, Servicebio, China), MMP2 (1:2,000, Servicebio, China), MMP9 (1:1,000, Affinity, China), SEMA3C (1:1,500, Affinity, China), smad2 (1:3,000, Affinity, China), p-smad2 (1:2,000, Affinity, China), smad3 (1:3,000, Affinity, China), p-smad3 (1:1,000, Affinity, China) and GAPDH (1:2,500, Servicebio, China), were incubated with the membranes overnight at 4°C. A secondary antibody (1:20,000, Servicebio, China) was incubated with the membrane for 90 min. The protein bands were developed with an enhanced chemiluminescence (ECL) kit (Meilunbio, China).

### *In vivo* experiment

The IA rats were established as described in the “*IA rats establishment*” subsection. Twenty-five male Sprague−Dawley (SD) rats (6–8 weeks old, 200–205 g, SLAC, China) were housed under specific pathogen-free conditions with 12/12 h light/dark cycles. Twenty SD rats were subjected to IA and classified into 4 groups: the model group (*n* = 5), which was stereotactically injected with 10 μL of PBS at the right basal cistern after osmotic pump implantation; the shMALAT1 group (*n* = 5), which was stereotactically injected with 10 μL of shMALAT1 lentivirus at the right basal cistern after osmotic pump implantation; the oeSEMA3C group (*n* = 5), which was stereotactically injected with 10 μL of oeSEMA3C lentivirus at the right basal cistern after osmotic pump implantation; and the shMALAT1 + oeSEMA3C group (*n* = 5), which was stereotactically injected with 10 μL of shMALAT1 lentivirus and oeSEMA3C lentivirus at the right basal cistern after osmotic pump implantation. Five SD rats that underwent the same surgical incisions without ligation or elastase injection were classified into the sham group. At 4 weeks after osmotic pump implantation, the serum and specimens were harvested, and the rats were sacrificed by intraperitoneal injection of pentobarbital sodium (150 mg/kg). The animal experiments were approved by the ethics committee (ethics approval no. 2023193-2). All procedures were performed according to the Guide for the Care and Use of Laboratory Animals.

### Hematoxylin—Eosin and immunohistochemistry staining

The IA tissues from the fixed rats were embedded in paraffin and cut into 4 μm slices. HE staining was performed with a Hematoxylin and Eosin Staining Kit (Servicebio, China) following the manufacturer’s protocols. For IHC staining, the slices were dewaxed, rehydrated, subjected to antigen retrieval, and blocked with endogenous peroxidase. The slices were subsequently incubated with primary antibodies overnight. The primary antibodies used were SEMA3C (1:200, Affinity, China), α-SMA (1:200, Servicebio, China), OPN (1:100, Servicebio, China), MMP2 (1:100, Servicebio, China), MMP9 (1:100, Affinity, China), p-smad2 (1:100, Affinity, China), and p-smad3 (1:150, Affinity, China). Afterward, the slices were incubated with secondary antibody (1:1,000, Servicebio, China) for 60 min. A 3,3’-diaminobenzidine (DAB) kit and hematoxylin were used to visualize the slices. An inverted microscope (Motic, China) was used to analyze the slices. IHC staining was semi-quantitatively evaluated based on staining intensity and the proportion of positive cells. Staining intensity was scored as 0 (negative), 1 (weak), 2 (moderate), or 3 (strong). The percentage of positive cells was scored as 0 ( < 5%), 1 (5–25%), 2 (26–50%), 3 (51–75%), or 4 ( > 75%). The final IHC score was calculated by multiplying the intensity score by the percentage score.

### Reverse transcription–quantitative polymerase chain reaction

The total RNA of the VSMCs (harvested at 72 h after infection) and the IA tissues of the rats was extracted with RNA isolation reagent and reverse transcribed to complementary DNA with RT SuperMix for qPCR (Servicebio, China). qPCR was performed with SYBR Green qPCR Master Mix (Servicebio, China). The thermal procedures were as follows: 95°C, 30 s, 1 cycle; 95°C, 15 s, 60°C, 30 s, 40 cycles. The primer sequences (5’- > 3’) used are listed in Supplementary Table 1. The results were calculated via the 2^–Δ^ ^Δ^ *^Ct^* method, and GAPDH was used as the internal reference gene.

### ELISA

The supernatants of the VSMCs (harvested at 72 h after infection) and the rat serum were collected. Rat TNF-α, IL-1β, and IL-6 ELISA kits (mlbio, China) were used to assess the TNF-α, IL-1β, and IL-6 concentrations in the supernatant and serum. The kits’ instructions were strictly followed.

### Competing endogenous RNA network analysis

Target miRNAs of MALAT1 were predicted using ENCORI/starBase database^1^ and the top 50 miRNAs were screened out by the rank of target-directed microRNA degradation (TDMD) score (Li et al., 2014). Moreover, Target miRNAs of SEMA3C were also predicted using ENCORI/starBase database, and the top 50 miRNAs were screened out by the rank of TDMD score. Then via cross analysis, the overlapped miRNAs were selected, and a lncRNA-mRNA-mRNA ceRNA network was proposed.

### Luciferase reporter assay

Predicted binding sequences of MALAT1 or SEMA3C were cloned into a luciferase reporter vector (wild-type, WT), and corresponding mutant constructs (MUT) with disrupted miR-944 seed regions were generated. HEK293T cells were co-transfected with WT or MUT reporters and miR-944 mimic or negative control miRNA (miR-NC) (Genechem, China). Luciferase activity was measured 48 h post-transfection using a luciferase reporter assay system (Beyotime, China).

### Statistical analysis

GraphPad 9.0 software (GraphPad, United States) was used for data analysis. The *in vitro* experiments were performed in triplicate (*n* = 3 in each group), and the *in vivo* experiments were performed in five replicates (*n* = 5 in each group). The data were presented as the means ± standard deviations. Differences among groups were compared by Tukey’s multiple tests. *P* < 0.05 was considered to indicate statistical significance.

## Results

### MALAT1 inhibited cell proliferation while facilitating the apoptosis and invasion of VSMCs

**FIGURE 1:**
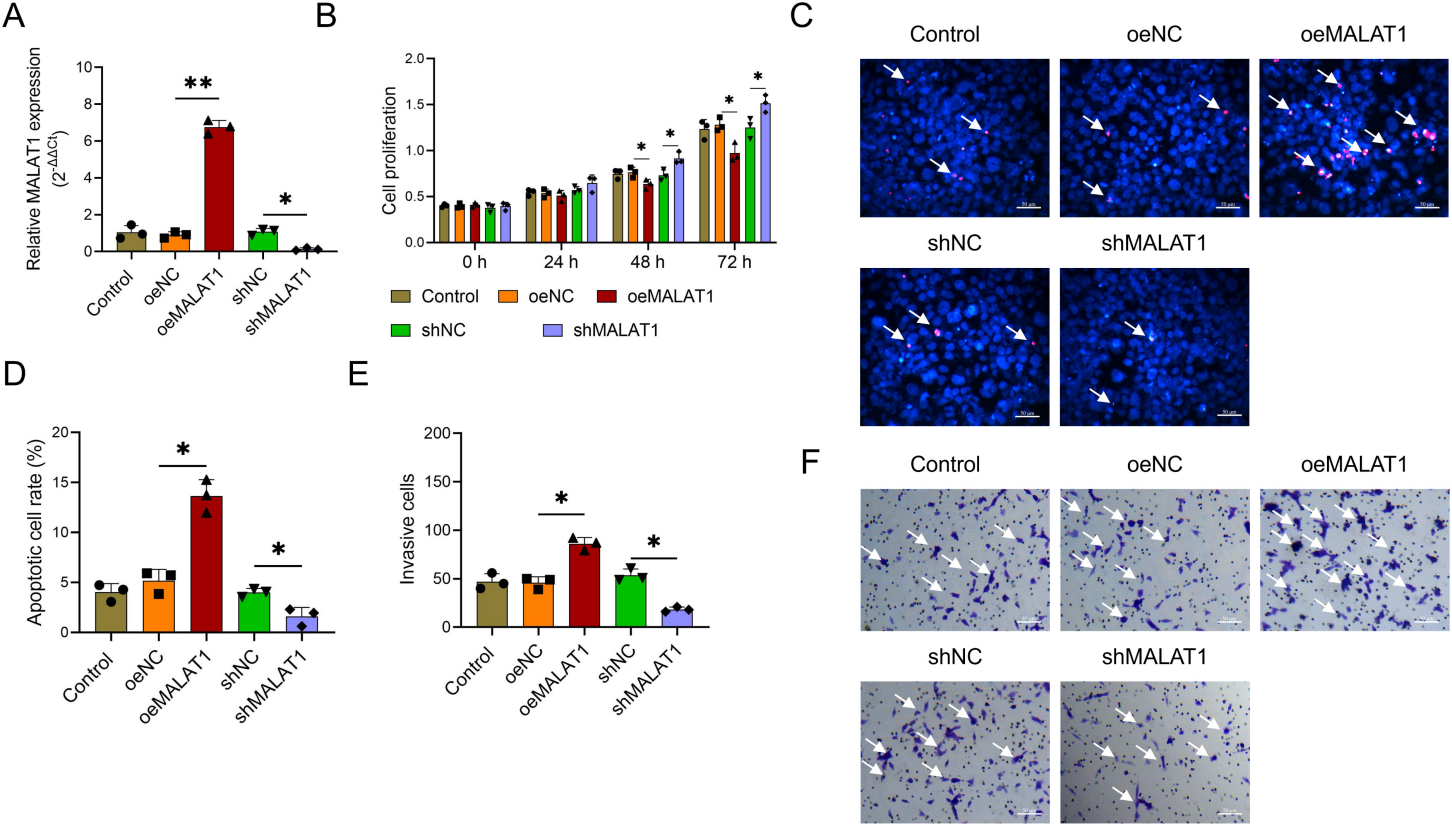
The effect of MALAT1 modification on the phenotype of VSMCs. The effects of MALAT1 modification on MALAT1 expression **(A)**, cell proliferation **(B)**, the apoptosis rate **(C,D)**, and the invasion rate **(E,F)** of VSMCs. Cell proliferation was detected via the CCK-8 assay. The apoptotic cell rate was detected via the TUNEL assay. The invasion rate was detected via a transwell assay. The arrow in **(C)** indicates a cell with red fluorescence, indicating an apoptotic cell. The arrow in **(F)** indicates the presence of crystalline purple spots, indicating invading cells. *P < 0.05, **P < 0.01.

To explore the effects of MALAT1 on the proliferation, apoptosis, and invasion of VSMCs, oeMALAT1 lentivirus (*P* < 0.01) and shMALAT1 lentivirus (*P* < 0.05) were successfully transfected into VSMCs (Figure 1A). The proliferation rate of VSMCs was lower at 48 h and 72 h in the oeMALAT1 group than in the oeNC group (both *P* < 0.05, Figure 1B). In contrast, the rates of cell apoptosis and invasion were greater in the oeMALAT1 group than in the oeNC group (both *P* < 0.05, Figures 1C–F). Furthermore, compared with oeMALAT1 lentivirus, shMALAT1 lentivirus had the opposite effect (all *P* < 0.05, Figures 1B–F). These findings indicated that MALAT1 inhibited cell proliferation while facilitating the apoptosis and invasion of VSMCs.

### MALAT1 promoted the release of inflammatory cytokines from VSMCs, which transformed from the contractile type to the synthetic type

**FIGURE 2:**
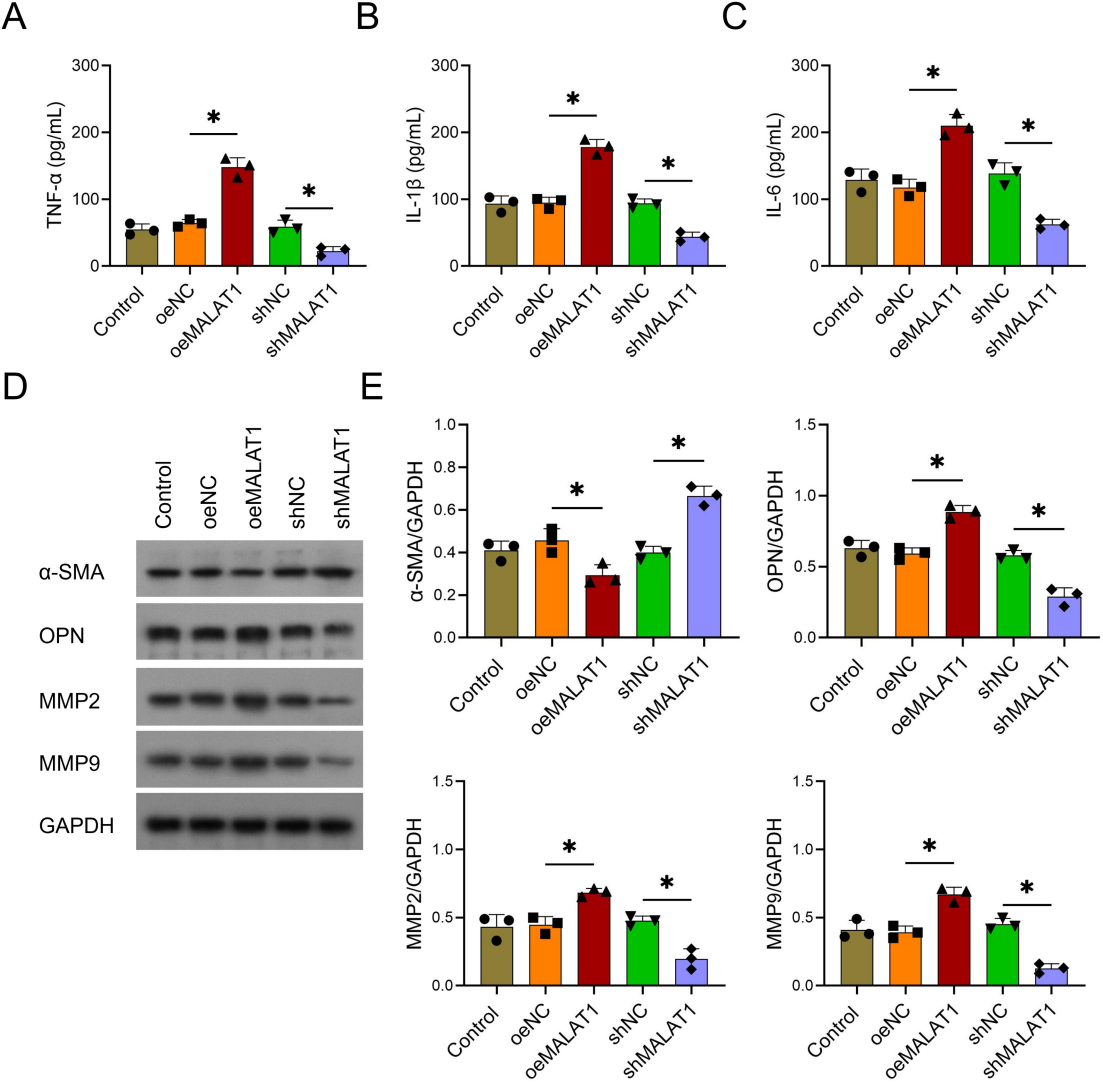
The effects of MALAT1 modification on inflammatory cytokines and phenotypic switching in VSMCs. Effects of MALAT1 modification on TNF-α **(A)**, IL-1β **(B)**, and IL-6 **(C)**, α-SMA, OPN, MMP2, and MMP9 protein expression **(D,E)**. TNF-α, IL-1β, and IL-6 were detected via ELISA. The detection of α-SMA, OPN, MMP2, and MMP9 was carried out via Western blot. *P < 0.05.

The levels of TNF-α, IL-1β, and IL-6 were greater in the oeMALAT1 group than in the oeNC group (all *P* < 0.05, Figures 2A–C), whereas the levels of these inflammatory cytokines were lower in the shMALAT1 group than in the shNC group (all *P* < 0.05, Figures 2A–C). These findings suggest that MALAT1 promotes the secretion of inflammatory cytokines in VSMCs. In addition, α-SMA was downregulated, whereas OPN, MMP2, and MMP9 were upregulated in the oeMALAT1 group compared with the oeNC group (all *P* < 0.05, Figures 2D,E). However, the opposite trend was observed in the shMALAT1 group compared with the shNC group (all *P* < 0.05, Figures 2D,E). These results revealed that MALAT1 promoted VSMC transformation from the contractile type to the synthetic type.

### MALAT1 was the upstream regulator of SEMA3C

**FIGURE 3:**
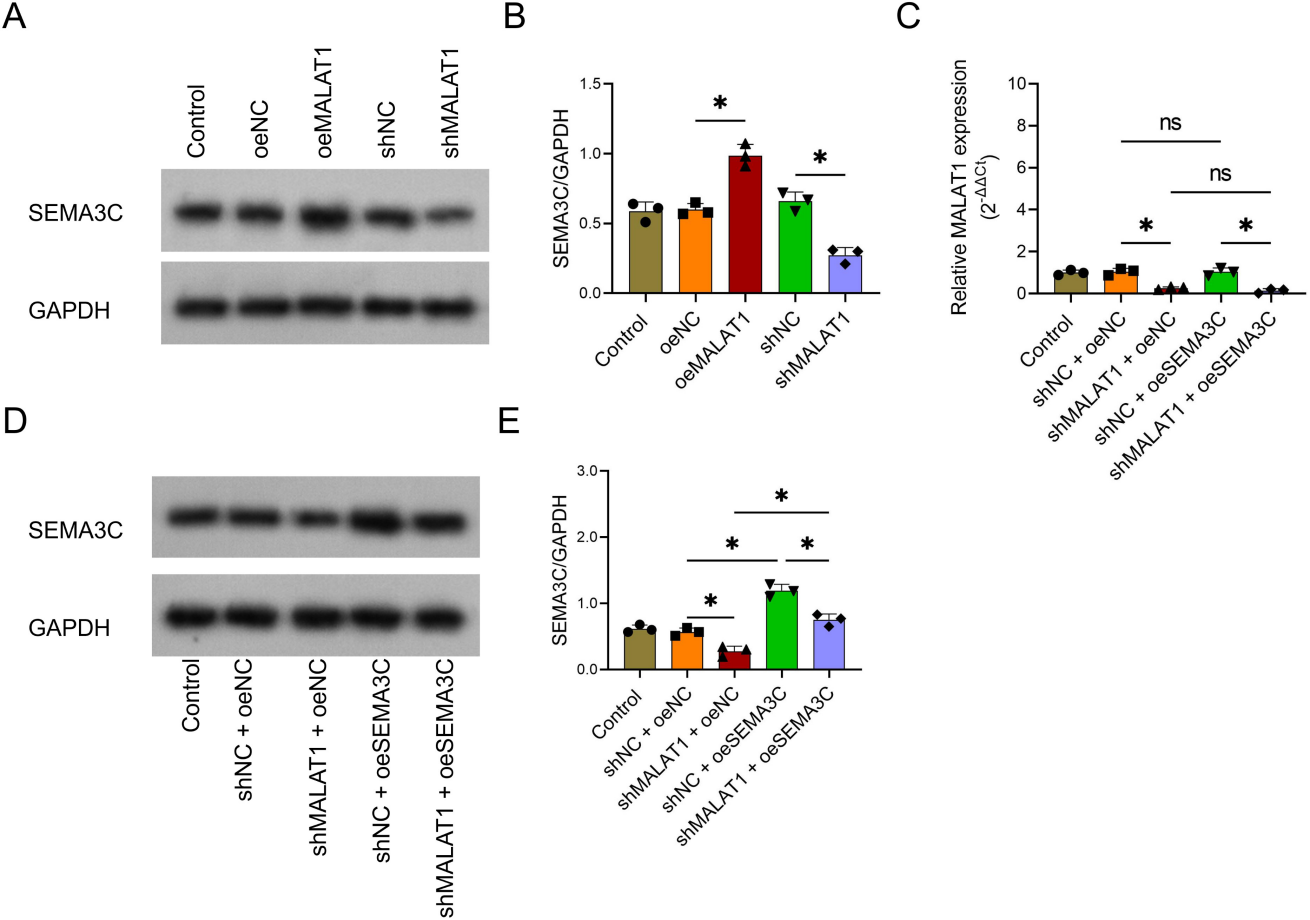
The effect of MALAT1 modification on SEMA3C expression. The effect of MALAT1 modification on SEMA3C protein expression **(A,B)**. Effects of MALAT1 silencing and SEMA3C overexpression on MALT1 expression **(C)** and SEMA3C expression **(D,E)**. *P < 0.05.

To explore the upstream and downstream relationships of SEMA3C and MALAT1, the oeMALAT1 lentivirus, shMALAT1 lentivirus, and oeSEMA3C lentivirus were transfected into VSMCs alone or in combination. SEMA3C was upregulated in the oeMALAT1 group compared with the oeNC group (*P* < 0.05) but downregulated in the shMALAT1 group compared with the shNC group (*P* < 0.05, Figures 3A,B). Interestingly, MALAT1 expression did not differ between the shNC+oeNC group and the shNC+oeSEMA3C group or between the shMALAT1+oeNC group and the shMALAT1+oeSEMA3C group (both *P* > 0.05, Figure 3C). Furthermore, SEMA3C in the shMALAT1+oeSEMA3C group was greater than that in the shMALAT1+oeNC group but lower than that in the shNC+oeSEMA3C group (both *P* < 0.05, Figures 3D,E). These findings indicate that MALAT1 is an upstream regulator of SEMA3C.

### Overexpression of SEMA3C attenuated the effects of MALAT1 on the proliferation, apoptosis, and invasion of VSMCs

**FIGURE 4:**
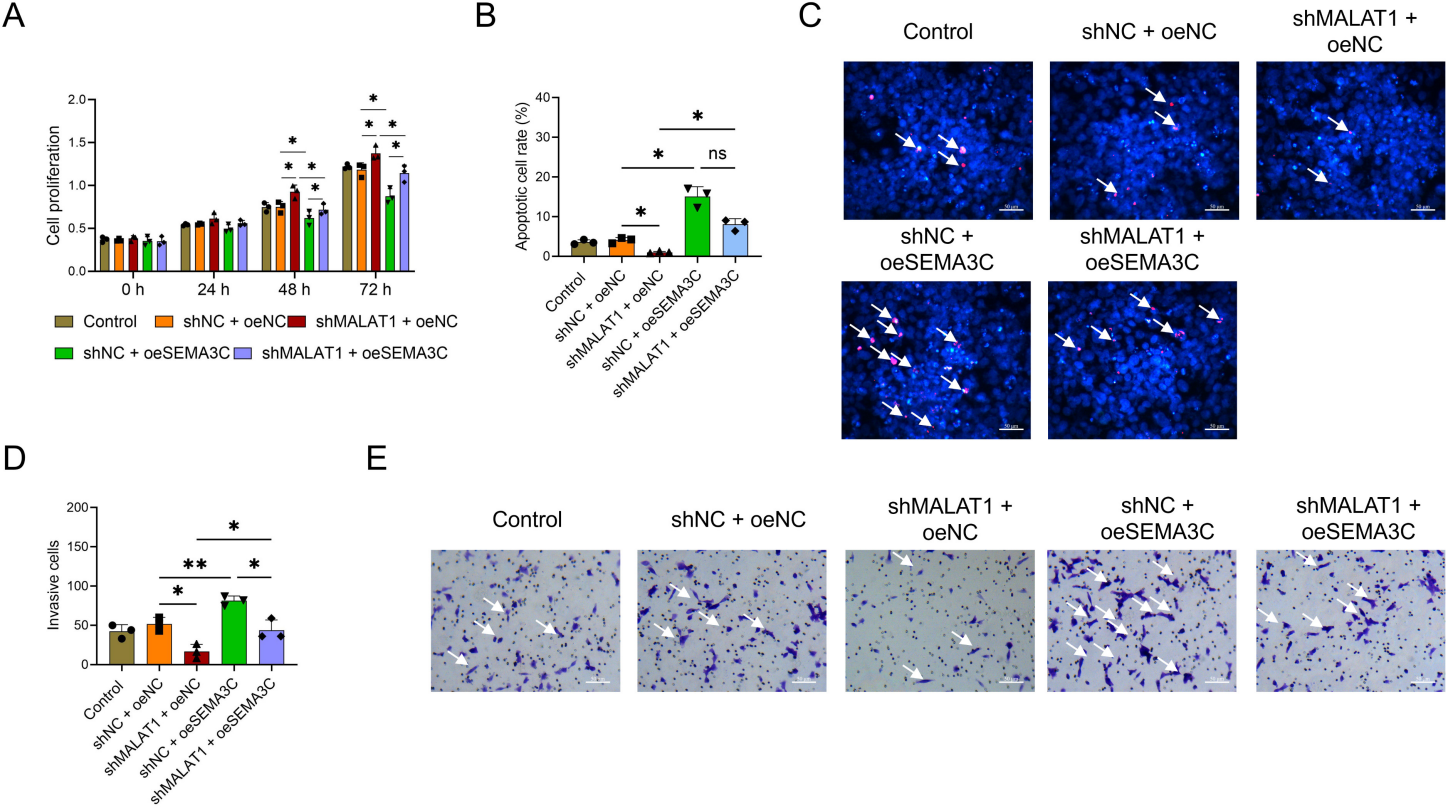
Overexpression of SEMA3C rescues the effects of MALAT1 on the proliferation, apoptosis, and invasion of VSMCs. Overexpression of SEMA3C rescued the effects of MALAT1 on the proliferation **(A)**, apoptosis **(B,C)**, and invasion rates **(D,E)** of VSMCs. The arrow in **(C)** indicates a cell with red fluorescence, indicating an apoptotic cell. The arrow in **(E)** indicates the presence of crystalline purple spots, indicating invading cells. *P < 0.05, **P < 0.01.

Cell proliferation was lower in the shNC+oeSEMA3C group than in the shNC+oeNC group, while it was greater in the shMALAT1+oeSEMA3C group than in the shNC+oeSEMA3C group at both 48 and 72 h (all *P* < 0.05, Figure 4A). In addition, the rates of apoptosis and invasion were greater in the shNC+oeSEMA3C group than in the shNC+oeNC group (both *P* < 0.05). The apoptosis rate did not significantly differ (*P* > 0.05), whereas the invasion rate was significantly lower in the shMALAT1+oeSEMA3C group than in the shNC+oeSEMA3C group (*P* < 0.05, Figures 4B–E).

### Overexpression of SEMA3C alleviated the effects of MALAT1 on the release of inflammatory cytokines and the transformation of cell types in VSMCs

**FIGURE 5:**
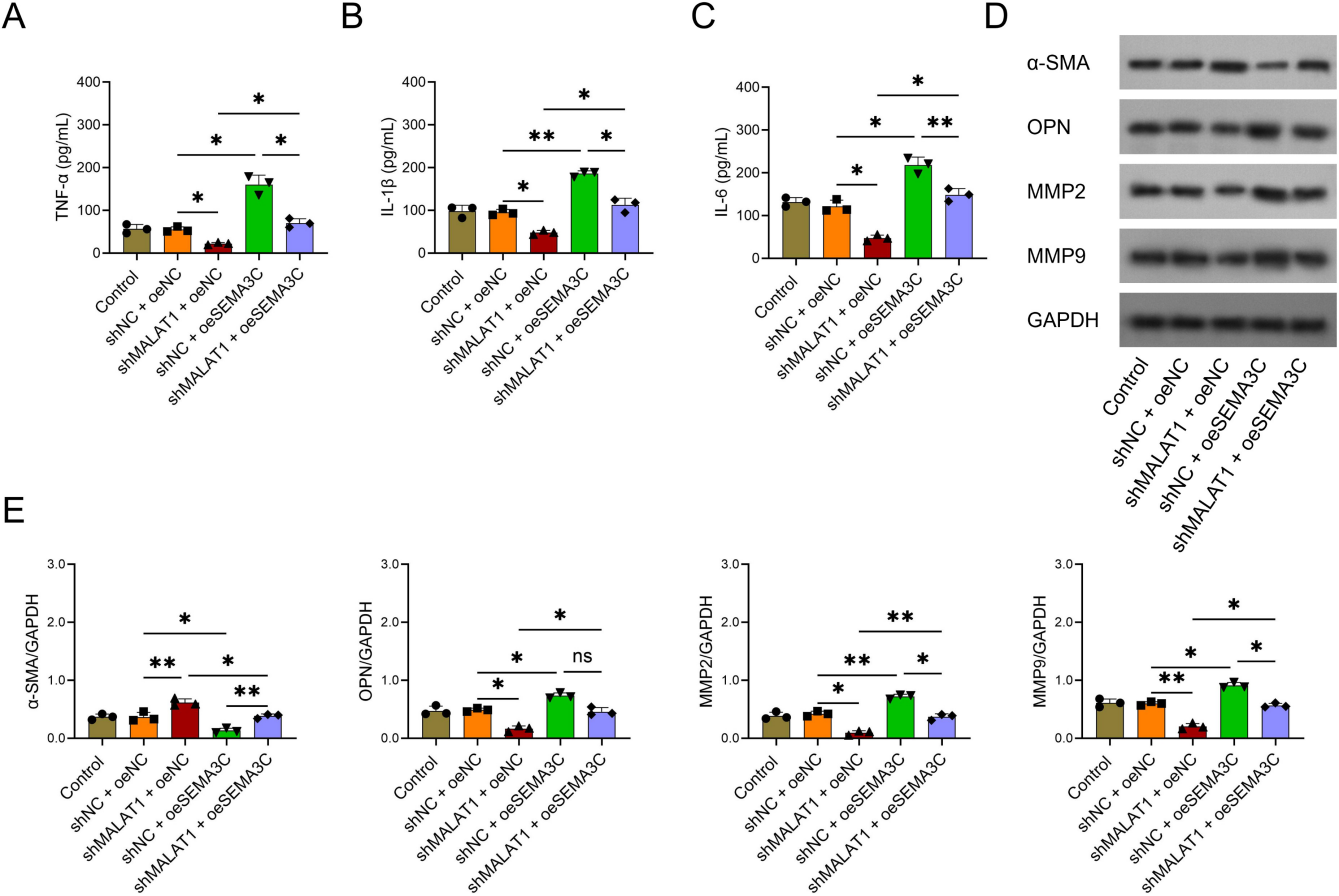
Overexpression of SEMA3C rescued the effects of MALAT1 on inflammatory cytokines and the phenotypic switch of VSMCs. Overexpression of SEMA3C rescued the effects of MALAT1 on TNF-α **(A)**, IL-1β **(B)**, and IL-6 **(C)**, α-SMA, OPN, MMP2, and MMP9 protein expression **(D,E)** in VSMCs. *P < 0.05, **P < 0.01.

*The levels of* TNF-α, IL-1β, and IL-6 were greater in the shNC+oeSEMA3C group than in the shNC+oeNC group (all *P* < 0.05, Figures 5A–C), whereas the levels of these inflammatory cytokines were lower in the shMALAT1+oeSEMA3C group than in the shNC+oeSEMA3C group (all *P* < 0.05, Figures 5A–C). These findings suggest that the overexpression of SEMA3C alleviated the effect of MALAT1 on the secretion of inflammatory cytokines in VSMCs. In addition, α-SMA was downregulated, whereas OPN, MMP2, and MMP9 were upregulated in the shNC+oeSEMA3C group compared with the shNC+oeNC group (all *P* < 0.05, Figures 5D,E). However, the opposite trend was observed in the shMALAT1+oeSEMA3C group compared with the shNC+oeSEMA3C group, except that OPN only tended to decrease without statistical significance (all *P* < 0.05, Figures 5D,E). These results revealed that the overexpression of SEMA3C decreased the ability of MALAT1 to promote VSMC transformation from the contractile type to the synthetic type.

### Silencing MALAT1 inactivates the Smad pathway via downregulation of SEMA3C

**FIGURE 6:**
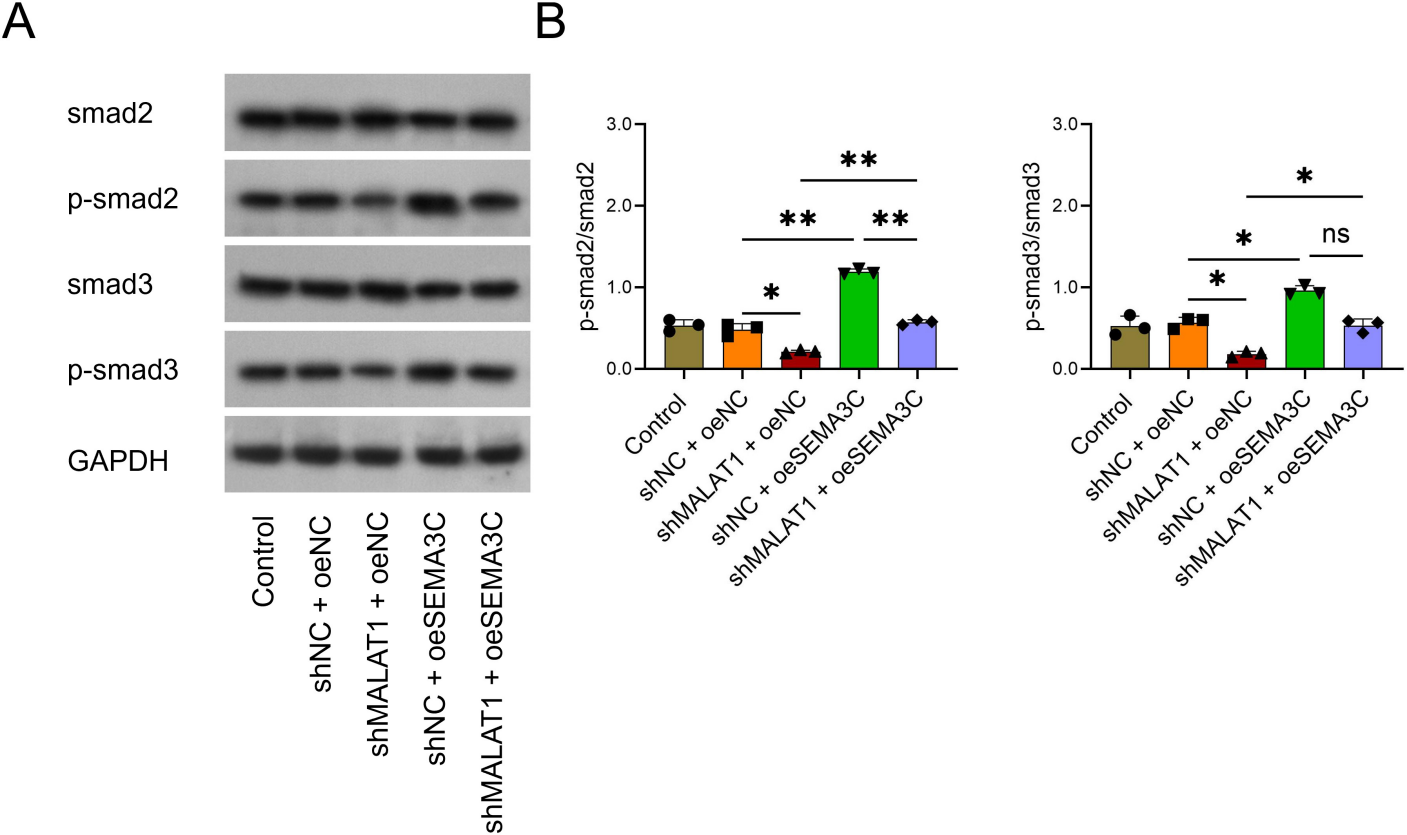
Overexpression of SEMA3C rescued the effect of MALAT1 on the Smad pathway in VSMCs. Overexpression of SEMA3C rescued the effects of MALAT1 on p-smad2 and p-smad3 **(A,B)**. *P < 0.05, **P < 0.01.

*The* p-smad2/smad2 and p-smad3/smad3 ratios were lower in the shMALAT1+oeNC group than in the shNC+oeNC group (both *P* < 0.05, Figures 6A,B). In addition, p-smad2/smad2 and p-smad3/smad3 were elevated in the shMALAT1+oeSEMA3C group compared with the shMALAT1+oeNC group (both *P* < 0.05, Figures 6A,B). These findings implied that downregulation of MALAT1 could inactivate the Smad pathway, whereas overexpression of SEMA3C could alleviate this inactivation.

### MALAT1 silencing reduced the release of proinflammatory cytokines, decreased IA formation, inhibited the transformation of the VSMC phenotype, and inactivated the SEMA3C-mediated Smad pathway in IA rats

**FIGURE 7:**
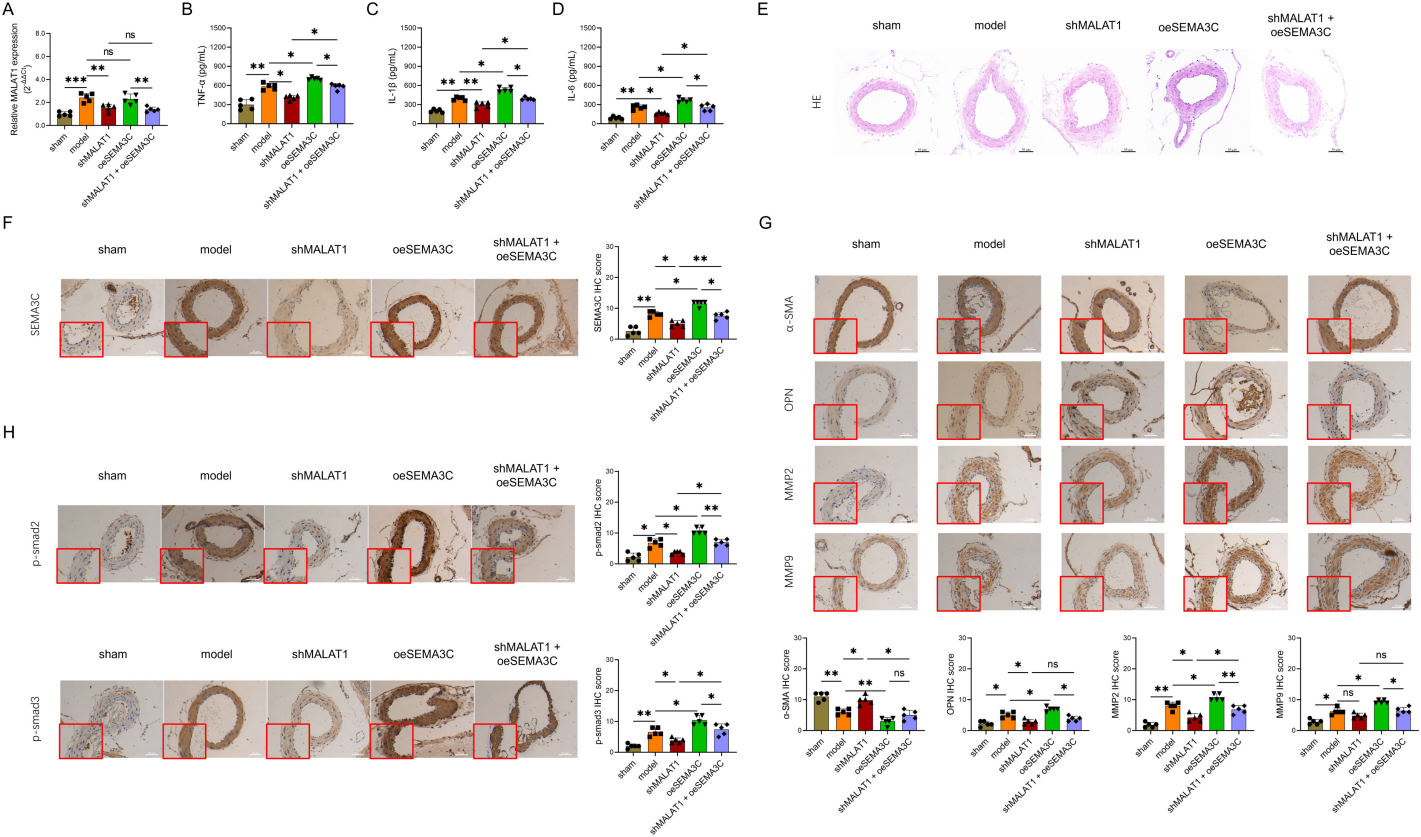
The effects of targeted MALAT1 on proinflammatory cytokines, IA formation, VSMC transformation, and the Smad pathway in IA rats. Effects of coinjection of MALAT1-silenced and SEMA3C-overexpressing lentiviruses on relative MALAT1 expression in IA tissue **(A)**, serum TNF-α **(B)**, serum IL-1β **(C)**, and serum IL-6 levels **(D)**, histological changes **(E)**, SEMA3C IHC scores **(F)**, α-SMA IHC scores, OPN IHC scores, MMP2 IHC scores, MMP9 IHC scores **(G)**, p-smad2 IHC scores and p-smad3 IHC scores **(H)**. *P < 0.05, **P < 0.01, ***P < 0.001.

After transfection with the lentiviral vector carrying fluorescent marker gene, a clear fluorescent signal was observed in the IA vessel wall area of the rats, indicating that the lentivirus was successfully infected (Supplementary Figure 1). Moreover, MALAT1 expression was decreased in the shMALAT1 group compared with the model group (*P* < 0.01, Figure 7A). In addition, the injection of oeSEMA3C lentivirus did not alter MALAT1 expression in the shMALAT1 lentivirus-treated rat model (*P* > 0.05, Figure 7A).

TNF-α, IL-1β, and IL-6 levels were lower in the shMALAT1 group than in the model group (all *P* < 0.05, Figures 7B–D), whereas these inflammatory cytokines were higher in the shMALAT1+oeSEMA3C group than in the shMALAT1 group (all *P* < 0.05, Figures 7B–D). These findings suggest that the overexpression of SEMA3C alleviated the effect of MALAT1 on the secretion of inflammatory cytokines in IA rats.

The HE staining image findings revealed that: The arterial wall structure in the Sham group was intact, with regular arrangement of smooth muscle cells and clear vascular wall layers. In contrast, in the model group, the arterial wall structure was disordered, the arrangement of smooth muscle cells was loose, and there were varying degrees of morphological abnormalities. In addition, compared with the model group, the arterial wall structure in the shMALAT1 group was less damaged, with reduced local vascular wall thinning and dilation. Compared with the Model group, the oeSEMA3C group showed further destruction of the arterial wall structure, with a looser arrangement of smooth muscle cells and a trend of cyst-like dilation in some areas. Finally, compared with the shMALAT1 group, the shMALAT1+oeSEMA3C group had more severe damage to the vascular wall structure, presenting disordered vascular wall layers and local thinning of the wall (Figure 7E).

In addition, the SEMA3C, OPN, MMP2, p-smad2, and p-smad3 levels were lower, whereas the α-SMA level was greater in the shMALAT1 group than in the model group (all *P* < 0.05, Figures 7F–H). Compared with that in the model group, the level of MMP9 in the shMALAT1 group tended to decrease, but the difference was not statistically significant (*P* > 0.05, Figure 7G). However, after the injection of the oeSEMA3C lentivirus, the SEMA3C, MMP2, p-smad2, and p-smad3 levels were greater in the shMALAT1+oeSEMA3C group than in the shMALAT1 group (Figures 7F–H). OPN and MMP9 only tended to increase, whereas α-SMA tended to decrease in the shMALAT1+oeSEMA3C group compared with the shMALAT1 group, but the difference was not statistically significant (all *P* > 0.05, Figure 7G).

**FIGURE 8:**
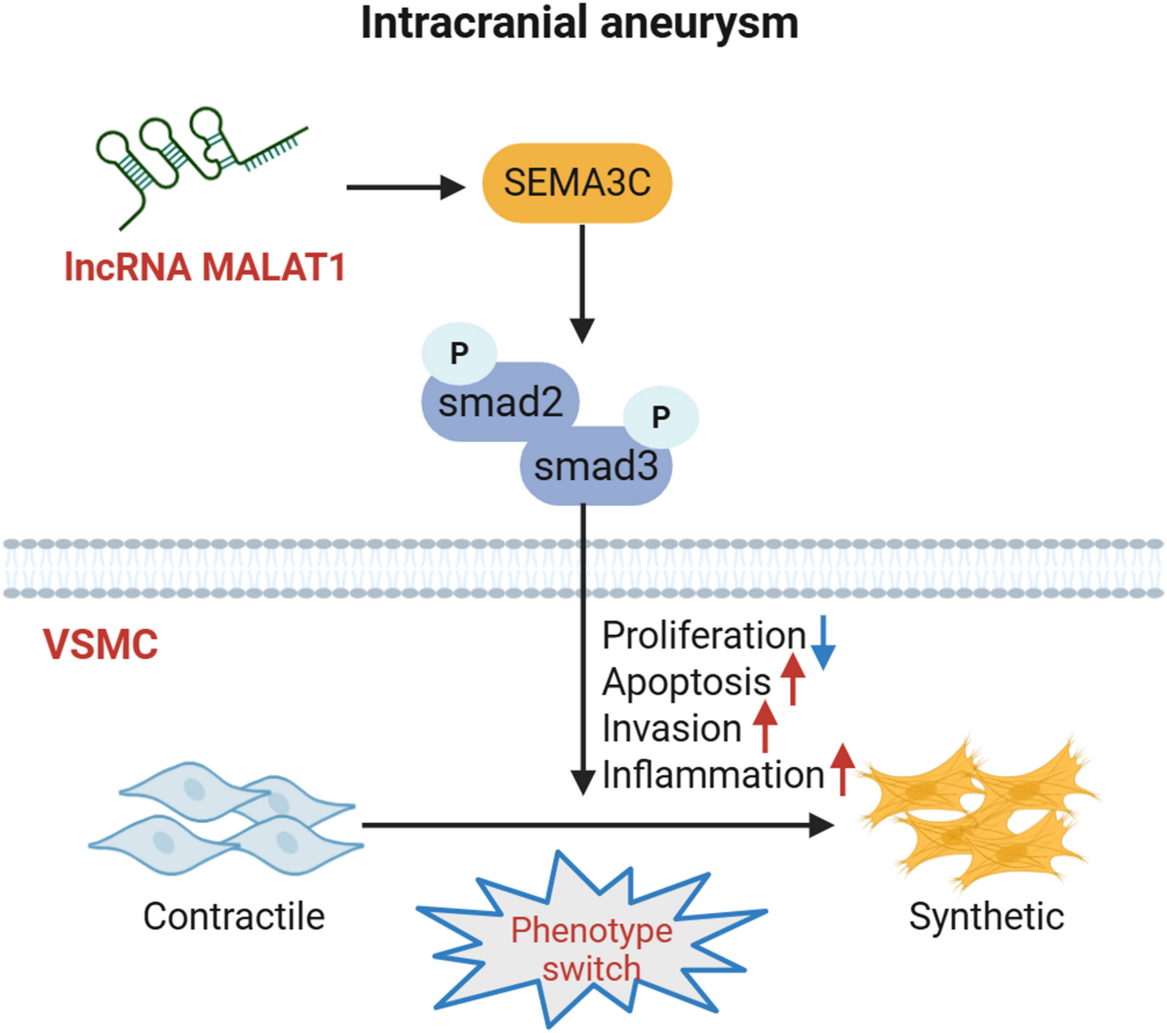
The hypothesized mechanism in this study.

These findings indicated that silencing MALAT1 reduced the release of proinflammatory cytokines, decreased IA formation, inhibited the VSMC phenotype switch, and inactivated the SEMA3C-mediated Smad pathway in IA rats. The hypothesized mechanism is illustrated in Figure 8.

### Regulatory relation between MALAT1 and SEMA3C

Via the prediction of the respective top 50 target miRNAs to MALAT1 and SEMA3C, followed by cross analysis, an overlapped miRNA was identified (miR-944) (Supplementary Figures 2A,B); therefore, a potential MALAT1-miR-944-SEMA3C ceRNA network was proposed. Then the luciferase reporter gene experiments were performed, which revealed that MALAT1 bound to miR-944, and miR-944 bound to SEMA3C, supporting the existence of a MALAT1-miR-944-SEMA3C ceRNA network (Supplementary Figure 2C).

## Discussion

MALAT1 has been reported to be a critical noncoding RNA implicated in various complex diseases, such as cancers, autoimmune diseases, vascular diseases, and neurological diseases (Khoshnam et al., 2024; Malakoti et al., 2023; Mohan et al., 2024; Puthanveetil et al., 2020), indicating its importance in human biological processes and functions. However, its function in IA has not been reported. This study revealed that MALAT1 overexpression increased cell apoptosis and invasion but decreased the proliferation of IA basilar artery VSMCs. In addition, the silencing of MALAT1 had opposite effects. The explanations for these phenomena may be as follows: (1) MALAT1 inhibition reduces the expression of MMPs and the secretion of inflammatory cytokines to repress Abdominal Aortic Aneurysm (AAA) (Yu et al., 2022), indicating that MALAT1 may function in IA basilar artery VSMCs as it functions in AAA. In addition, this speculation was validated in our subsequent experiments. (2) MALAT1 promotes the progression of vessel atherosclerosis via the regulation of multiple signaling pathways, such as the mTOR pathway and NF-κB pathway (Shi et al., 2021; Zhu et al., 2019), via which MALAT1 may function in IA basilar artery VSMCs as it functions in the atherosclerotic process. (3) MALAT1 affects the growth, migration, and invasion of VSMCs via Nrf2–GPX4 signaling, miR-143, and the miR-124–3p.1–KLF5 axis (Cao et al., 2021; Liao et al., 2024; Wang et al., 2019); then, via the same mechanism, MALAT1 regulates IA basilar artery VSMCs. (4) MALAT1 upregulates SEMA3C and the downstream Smad pathway to increase cell apoptosis and invasion but reduces the proliferation of IA basilar artery VSMCs, as discovered in our subsequent experiments in this study.

This study revealed that MALAT1 overexpression induced the secretion of inflammatory cytokines in IA basilar artery VSMCs and that MALAT1 silencing repressed their levels. In addition, this study further confirmed that MALAT1 silencing decreased inflammation in IA rats. These findings suggest that targeting MALAT1 could suppress IA inflammation both *in vitro* and *in vivo*. Several possible explanations may explain these findings: (1) MALAT1 regulates several inflammation-related genes and proteins, such as miR-135b-5p, miR-361-3p, CXCR7, NLRP3, and GRP78 (Khoshnam et al., 2024; Malakoti et al., 2023; Mohan et al., 2024; Puthanveetil et al., 2020; Yu et al., 2022), to increase the secretion of inflammatory cytokines. (2) MALAT1 modifies several well-known inflammatory signaling pathways, such as the JNK pathway, NF-κB pathway, Wnt pathway, TLR4 pathway, and ERK-MAPK pathway (Gong et al., 2020; Li J. et al., 2020; Liu et al., 2024; Shi Y. L. et al., 2019), to increase the secretion of inflammatory cytokines. (3) MALAT1 modifies the SEMA3C-mediated Smad pathway to facilitate inflammation in IA basilar artery VSMCs and IA rats, as revealed by our subsequent experiments in this study.

α-SMA, OPN, MMP2, and MMP9 are common markers applied to evaluate the phenotype transformation of VSMCs (contractile or synthetic) (Mao et al., 2015; Zhu et al., 2015); therefore, they are chosen to detect in this study. By detecting markers of the VSMC phenotype, this study revealed that MALAT1 overexpression promoted the transformation of IA basilar artery VSMCs from the contractile type to the synthetic type, but MALAT1 silencing had the opposite effect on this transformation. Moreover, MALAT1 silencing reversed VSMC transformation from the contractile type to the synthetic type in IA rats. These findings suggested that targeting MALAT1 was able to reverse VSMC transformation from the contractile type to the synthetic type both *in vitro* and *in vivo*. The possible explanations may be as follows: (1) MALAT1 sponges miR-142-3p to modify ATG7 expression to regulate the phenotypic switch of VSMCs (Song et al., 2018). (2) MALAT1 regulates BRG1 and histone H3-lysine 27 trimethylation to modify contractile protein expression to regulate the phenotypic switch of VSMCs (Lino Cardenas et al., 2018). (3) The SEMA3C-mediated Smad pathway is involved in the regulatory effect of MALAT1 on the phenotypic switch of VSMCs, as shown in our subsequent experiments. It could be noted that even though MALAT1 promoted the transformation of IA basilar artery VSMCs from the contractile type to the synthetic type, but it repressed the VSMC proliferation, the possible explanations may be as follows: (1) The origin of VSMCs and their pathological state may lead to the effect of MALAT1 on inhibiting proliferation. The VSMCs used in this study are isolated from primary IA basilar artery tissues in pathological conditions, and primary VSMCs from pathological sources have significant differences in gene expression profiles, phenotypic stability, and cell cycle regulation compared to normal or induced model VSMCs, whose proliferation ability and response to regulatory factors also exhibit significant heterogeneity (Hu et al., 2023). For instance, a previous study reported that MALAT1 suppressed the proliferation of ox-LDL-treated VSMCs (Cheng et al., 2019). (2) Synthetic VSMCs in IA can be further subdivided into several subtypes with different functional preferences, including those mainly characterized by proliferation and migration, or inflammatory/secreting functions (Wang et al., 2023). During the stage of vascular injury and reconstruction, VSMCs often exhibit an enhanced migratory ability while their proliferation is temporarily inhibited, which facilitates cytoskeleton rearrangement and directed migration, reflecting the functional “decoupling” of migration and proliferation under certain conditions (Louis and Zahradka, 2010).

SEMA3C is an important neural guidance factor that belongs to the cysteine-enriched semaphorin family and regulates neural axon guidance and neural network formation, angiogenesis and vascular remodeling, immune responses and inflammation (Martins et al., 2022; Shi et al., 2022; Toyofuku et al., 2008; Vadivel et al., 2013; Yang et al., 2015). In addition, according to previous RNA-sequencing data, MALAT1 silencing strongly reduces the expression of SEMA3C (Wernig-Zorc et al., 2024). Therefore, the interaction between MALAT1 and SEMA3C was explored in this study. This study revealed that SEMA3C overexpression enhanced dysfunction, inflammation, and synthetic-type switching in IA basilar artery VSMCs and promoted pathological progression, inflammation, and synthetic-type switching in IA rats. Importantly, MALAT1 positively regulated SEMA3C expression, and SEMA3C overexpression reversed the effect of MALAT1 silencing on IA basilar artery VSMCs and IA rats, indicating its involvement in MALAT1 regulatory functions. The explanations for these phenomena may be as follows: (1) SEMA3C promotes VSMC invasion and phenotype switching via its interaction with GATA-6, FOXO1, and TGF-β (Lepore et al., 2006; Niimi et al., 2024), and it promotes inflammation via the NRP2-Hedgehog pathway (Yang et al., 2022), which facilitates the progression of IA. (2) SEMA3C is related to the FOXO1-TGFβ-Smad pathway [30], and the Smad pathway is an important signaling pathway implicated in the progression of arterial aneurysms (Da et al., 2023; Hu et al., 2020; Yuan, 2012); therefore, SEMA3C may promote the progression of IA via its activation of the Smad pathway.

The Smad pathway is a well-known signaling pathway that participates in the pathogenesis of various diseases, such as vascular diseases, neurological diseases, and inflammatory diseases (Hiew et al., 2021; Lan, 2011; Liu et al., 2023). Furthermore, MALAT1 and SEMA3C are both regulators of the Smad pathway (Niimi et al., 2024; Zhang et al., 2020). Therefore, this study further investigated whether the Smad pathway is regulated by MALAT1 and SEMA3C in IA. This study revealed that MALAT1 and SEMA3C both positively regulate the Smad pathway in IA basilar artery VSMCs and IA rats. Combining the interaction between MALAT1 and SEMA3C, a MALAT1-SEMA3C-Smad signaling network was proposed for IA. A possible explanation may result from the important function of the Smad pathway in regulating angiogenesis, vascular remodeling, and inflammation (Lan, 2011; Liu et al., 2023). In the aspect of relation between SEMA3C and Smad pathway, we consider it may be mediated by receptors. SEMA3C, as a secreted protein, usually functions on the cell membrane through Plexin/Neuropilin receptor (Lu et al., 2021), and the Plexin/Neuropilin receptor signaling is reported to be sequential or cross-regulated with the TGF-β signaling (Kwiatkowski et al., 2017). Additionally, by constructing a protein-protein interaction network using STRING database (Szklarczyk et al., 2023), it observes that SEMA3C may activate the Smad pathway (reflected by Smad2 and Smad3) through an indirect pathway mediated by the receptors (Supplementary Figure 3). Therefore, we speculate that SEMA3C may activate the Smad pathway by the mediation of the receptors, but the specific receptors and molecular mechanisms require further study.

In conclusion, silencing MALAT1 represses pathological progression, inflammation, and VSMC phenotype switching via its regulation of the SEMA3C-mediated Smad pathway in IA. This finding suggests the potency of MALAT1 as a target for IA treatment, which might improve the prognosis compared with the traditional treatment strategy, such as surgery or symptomatic treatment; however, further in-depth validations are needed in the future.

## Data Availability

The original contributions presented in the study are included in the article/Supplementary material, further inquiries can be directed to the corresponding authors.

## Footnotes

1
https://rnasysu.com/encori/

## Supplementary material

Supplementary Figure 1 Observation of the infected efficiency of lentivirus by fluorescent marker gene method.

Supplementary Figure 2 Relation between MALAT1 and SEMA3C. Venn diagram between the top 50 predicted miRNAs targeting MALAT1 and the top predicted miRNAs targeting SEMA3C **(A)**, ceRNA network between MALAT1 and SEMA3C **(B)**, and luciferase reporter gene experiments **(C)**.

Supplementary Figure 3 Protein-protein interaction analysis.
