# Supplementary material for: Silencing MALAT1 represses pathological progression, inflammation, and vascular smooth muscle cell phenotype switching by regulating the SEMA3C-mediated Smad pathway in intracranial aneurysms
Source: Front Cell Neurosci. 2026 Mar 11;20:1706518. doi: 10.3389/fncel.2026.1706518 (PMC13013063; doi:10.3389/fncel.2026.1706518)
Supplement: Supplementary file 2 [file Table_1.docx]

**Supplementary Table 1.** Primers’ information.

| Gene symbol | Forward primer (5‘-3’) | Reverse primer (5‘-3’) |
| --- | --- | --- |
| GAPDH | AAGTTCAACGGCACAGTCAAG | ACATACTCAGCACCAGCATCA |
| MALAT1 | CCTGCGGTGTCTTTGCTTGAC | CATACCCAGAGCCTTTAGAACCC |
