# Supplementary material for: Silencing MALAT1 represses pathological progression, inflammation, and vascular smooth muscle cell phenotype switching by regulating the SEMA3C-mediated Smad pathway in intracranial aneurysms
Source: Front Cell Neurosci. 2026 Mar 11;20:1706518. doi: 10.3389/fncel.2026.1706518 (PMC13013063; doi:10.3389/fncel.2026.1706518)

**Uncropped Western Blot images**

Figure 2D _α-SMA


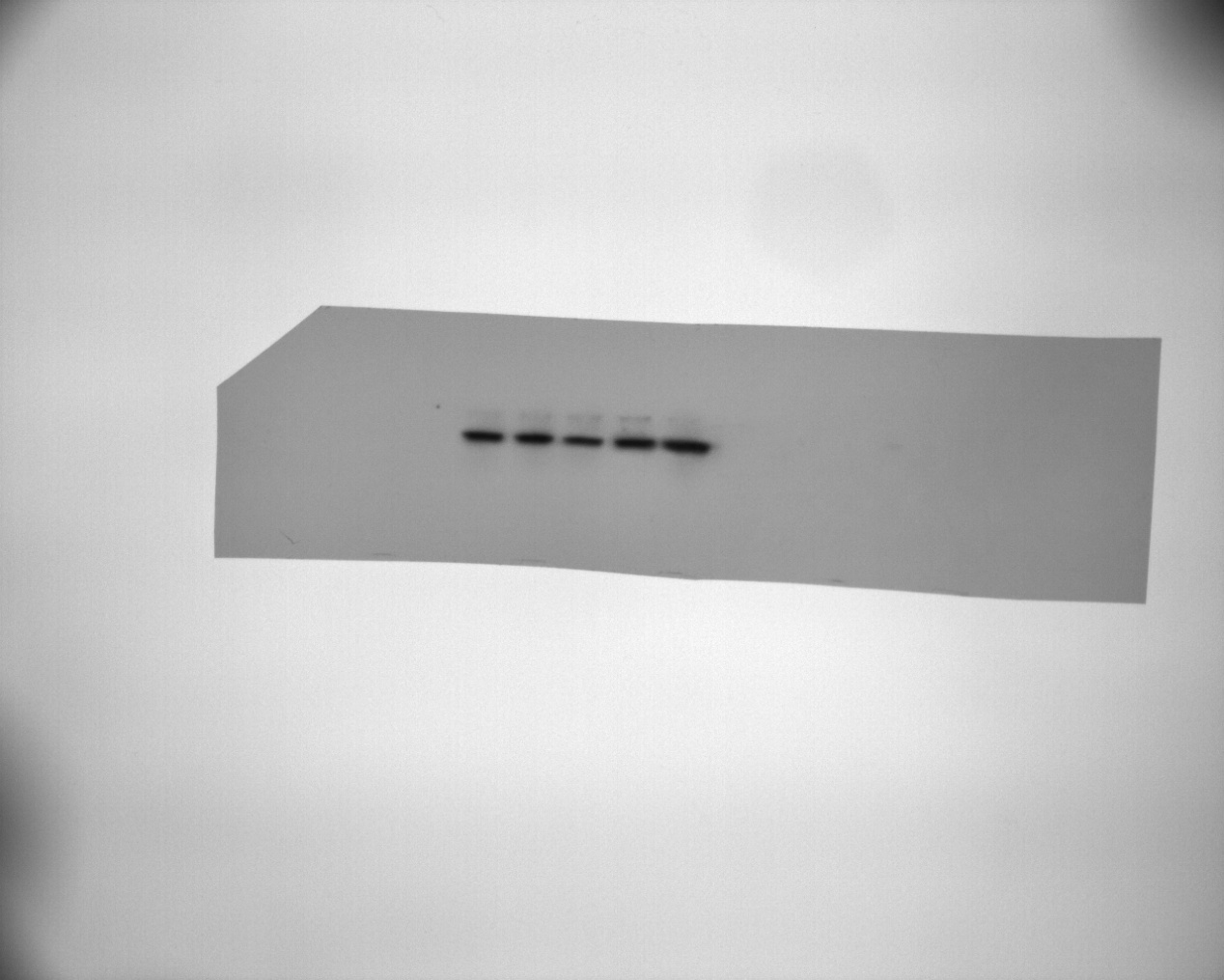


Figure 2D _OPN


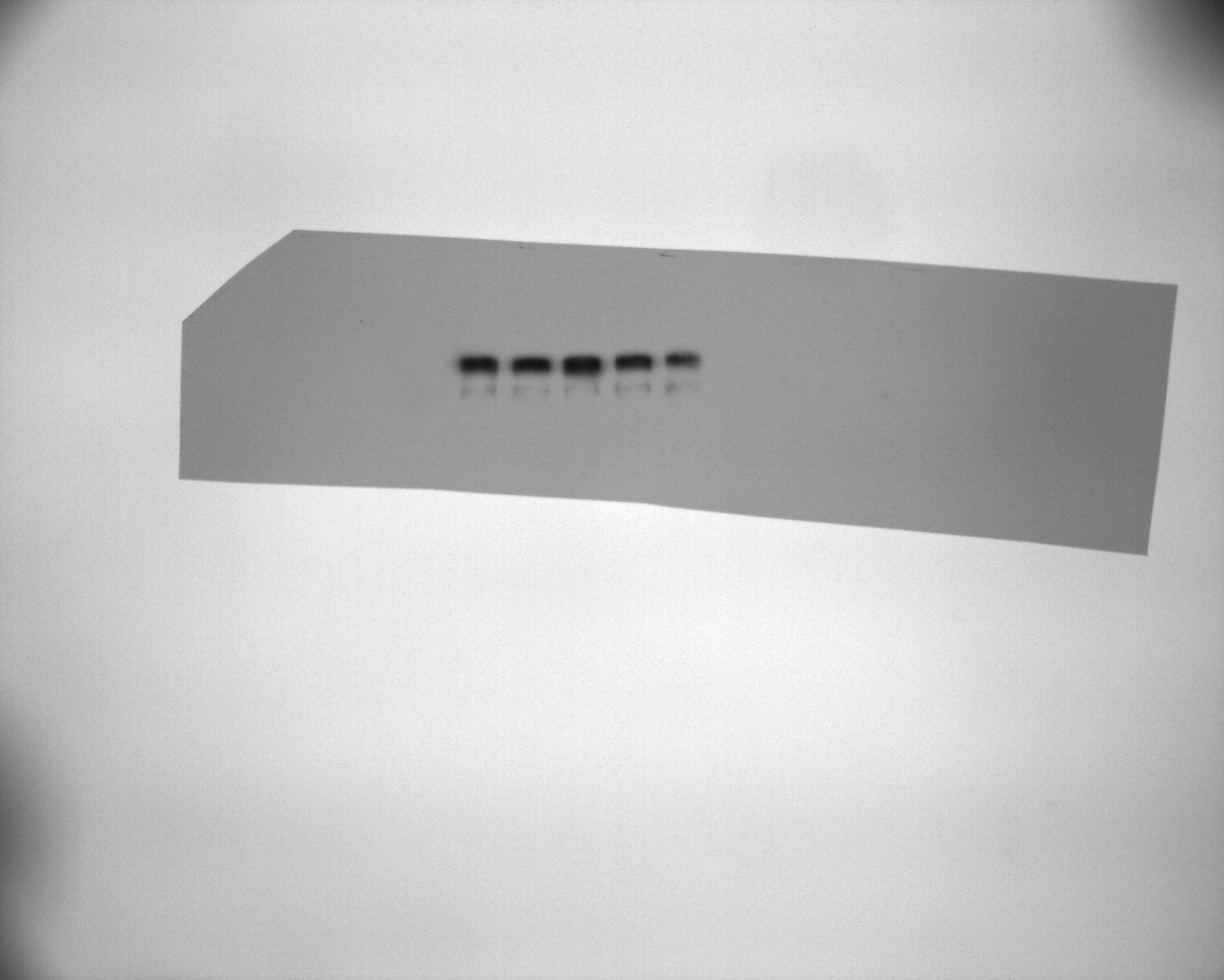


Figure 2D _MMP2


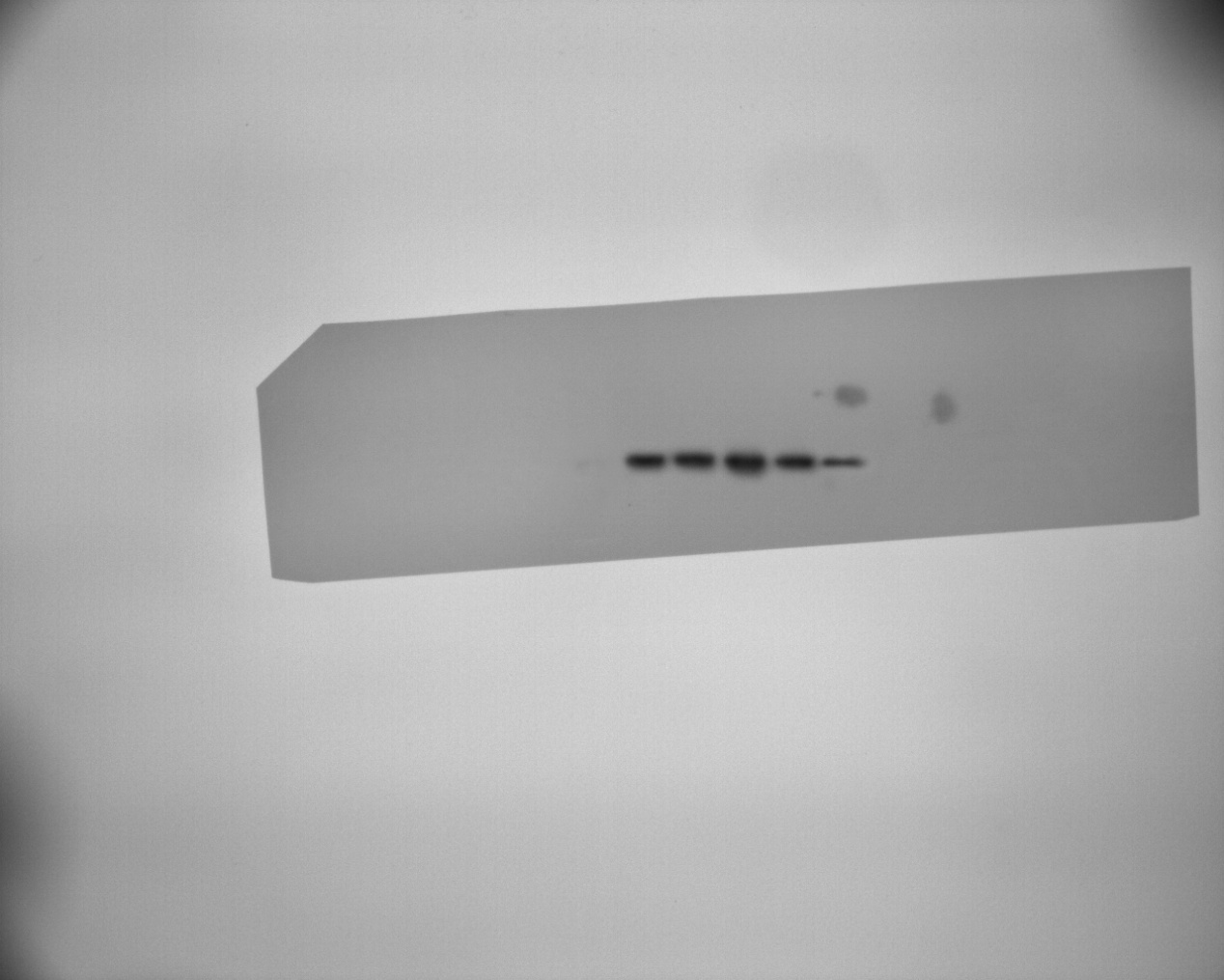


Figure 2D _MMP9


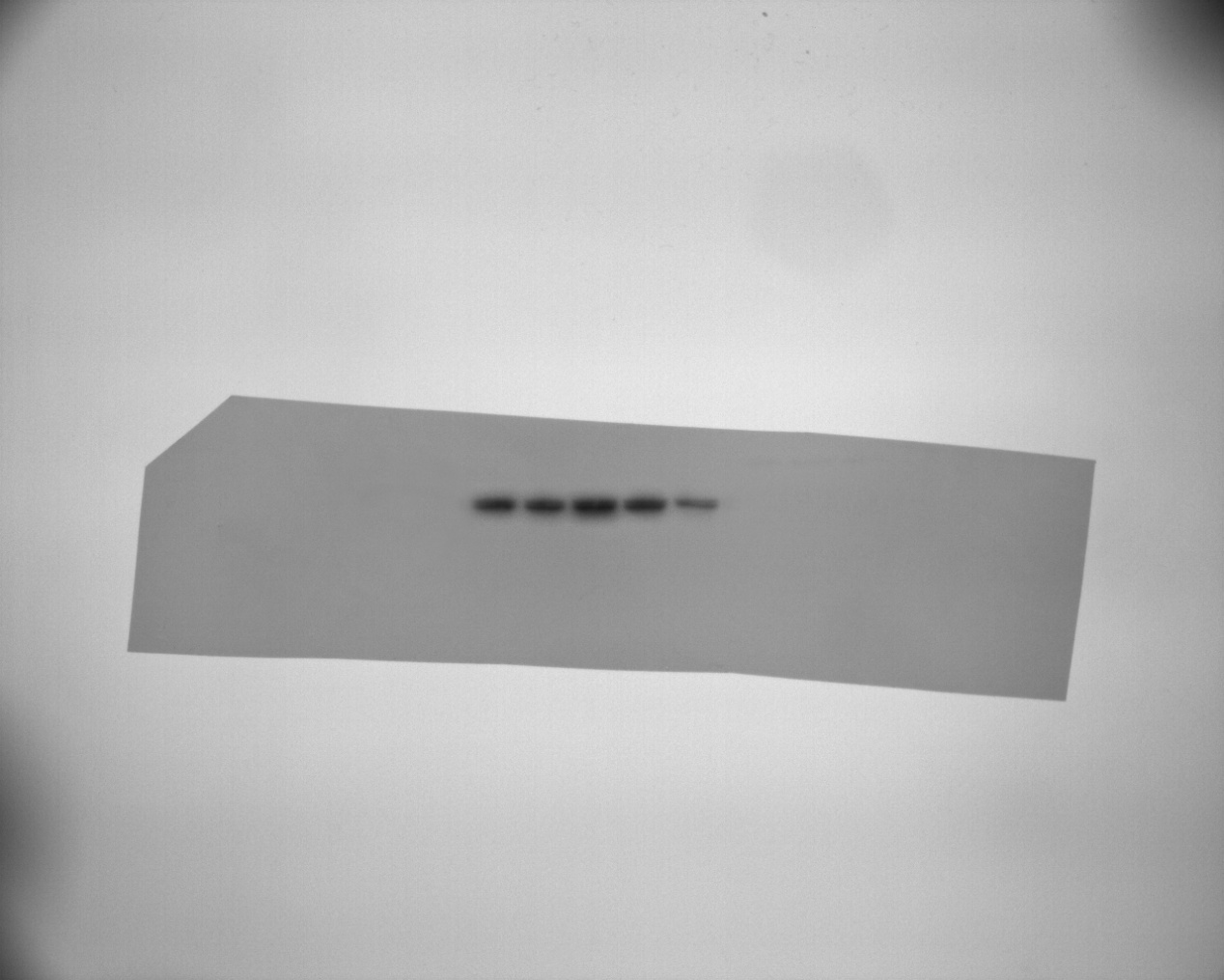


Figure 2D _GAPDH


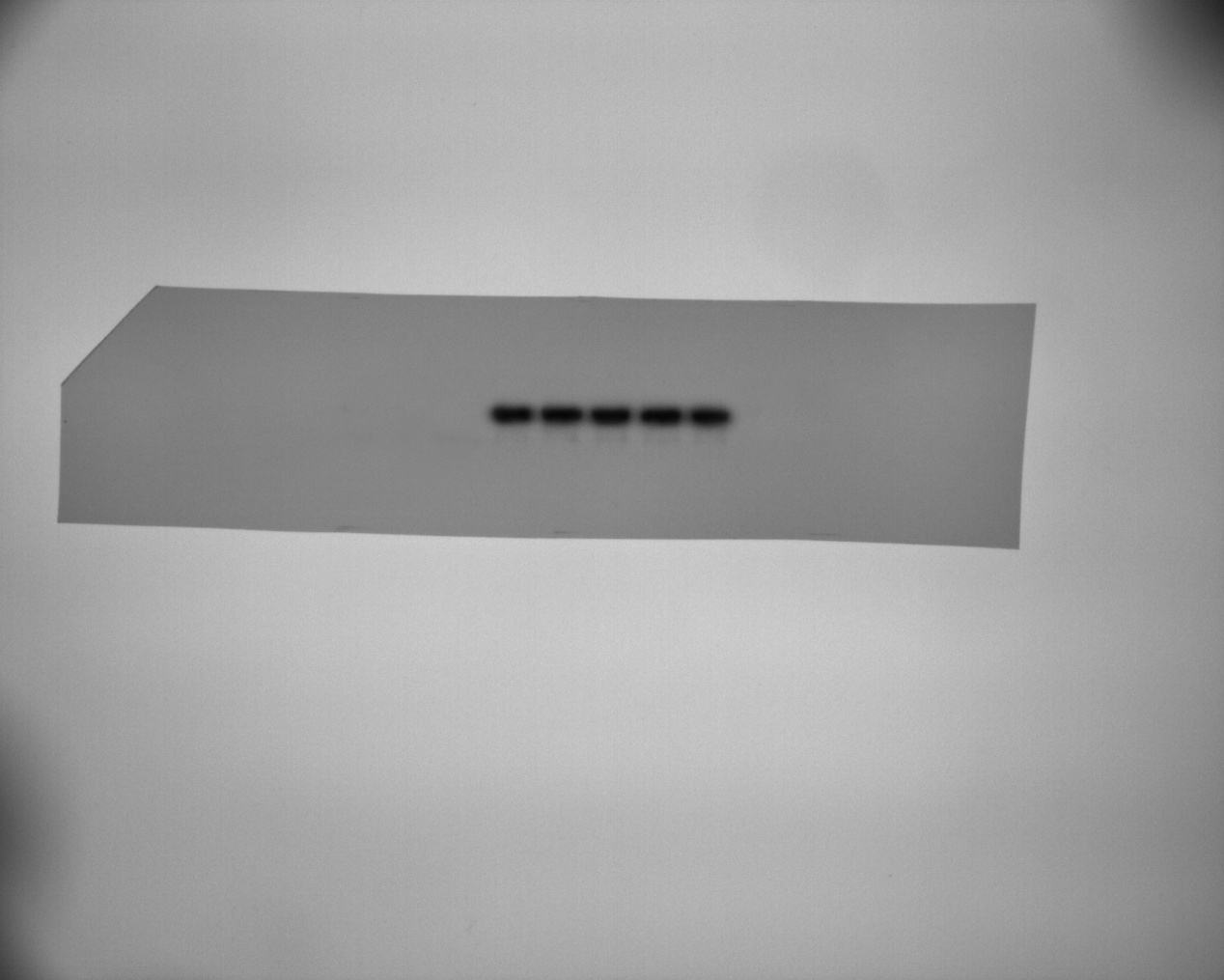


Figure 3A _SEMA3C


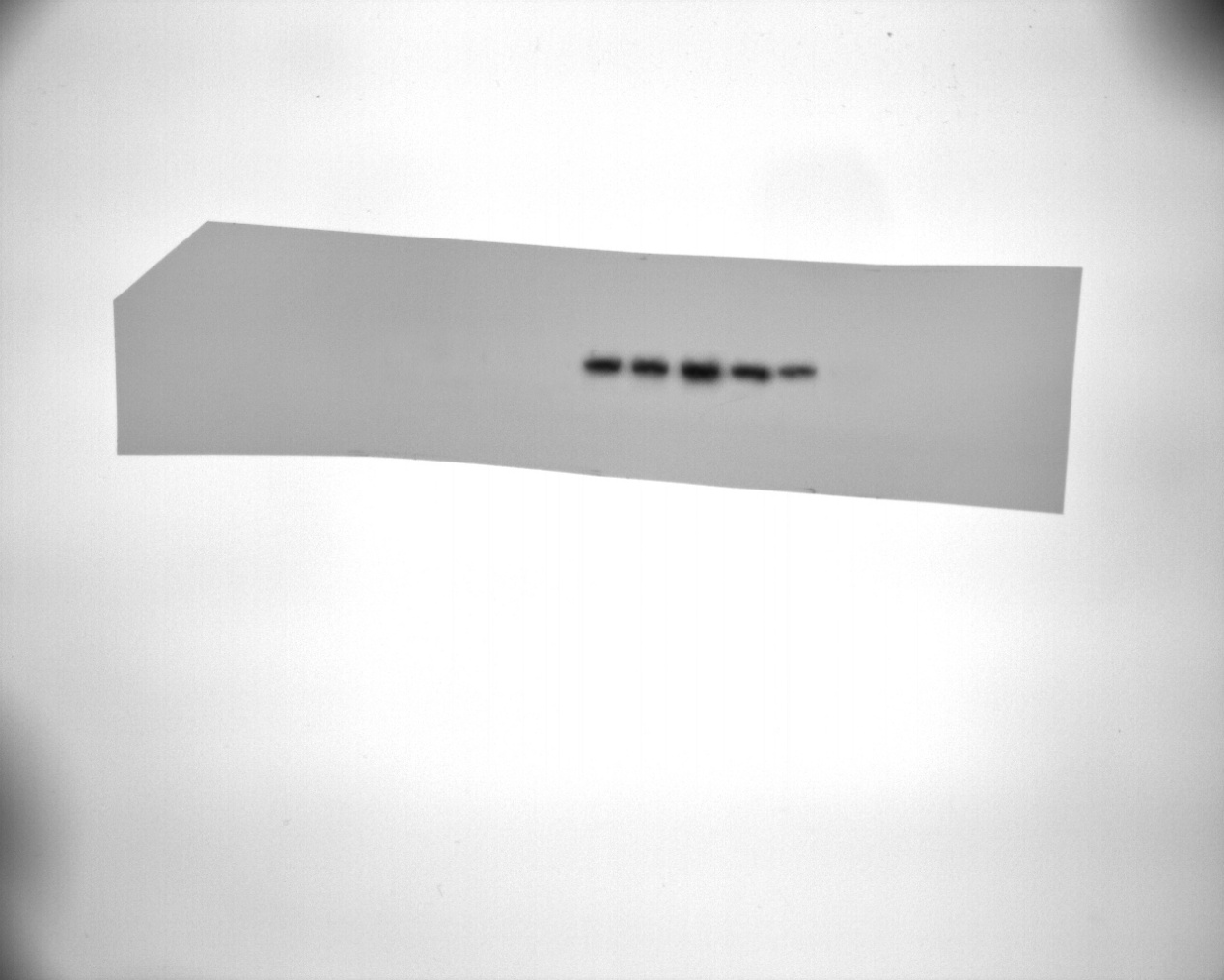


Figure 3A _GAPDH


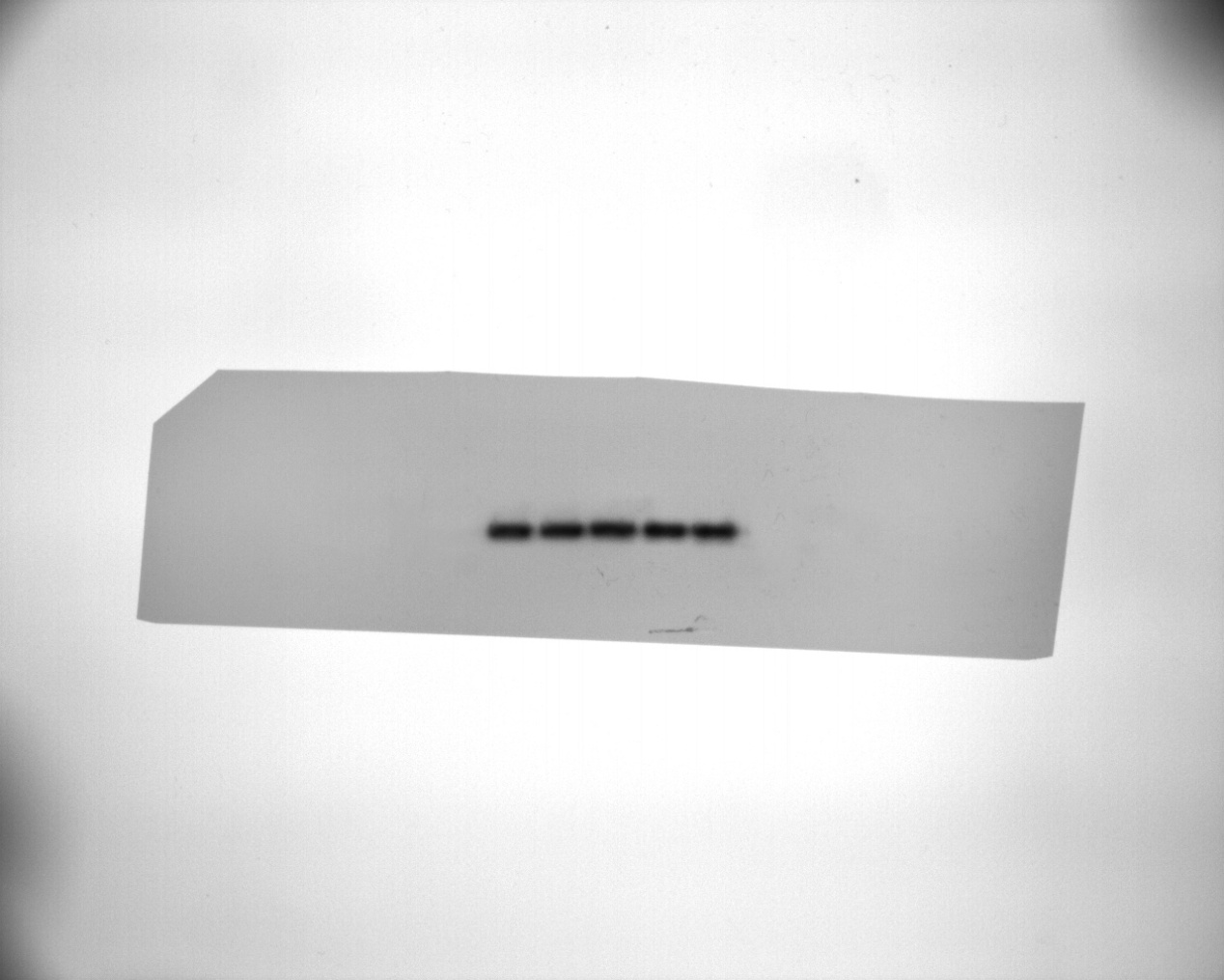


Figure 3D _SEMA3C


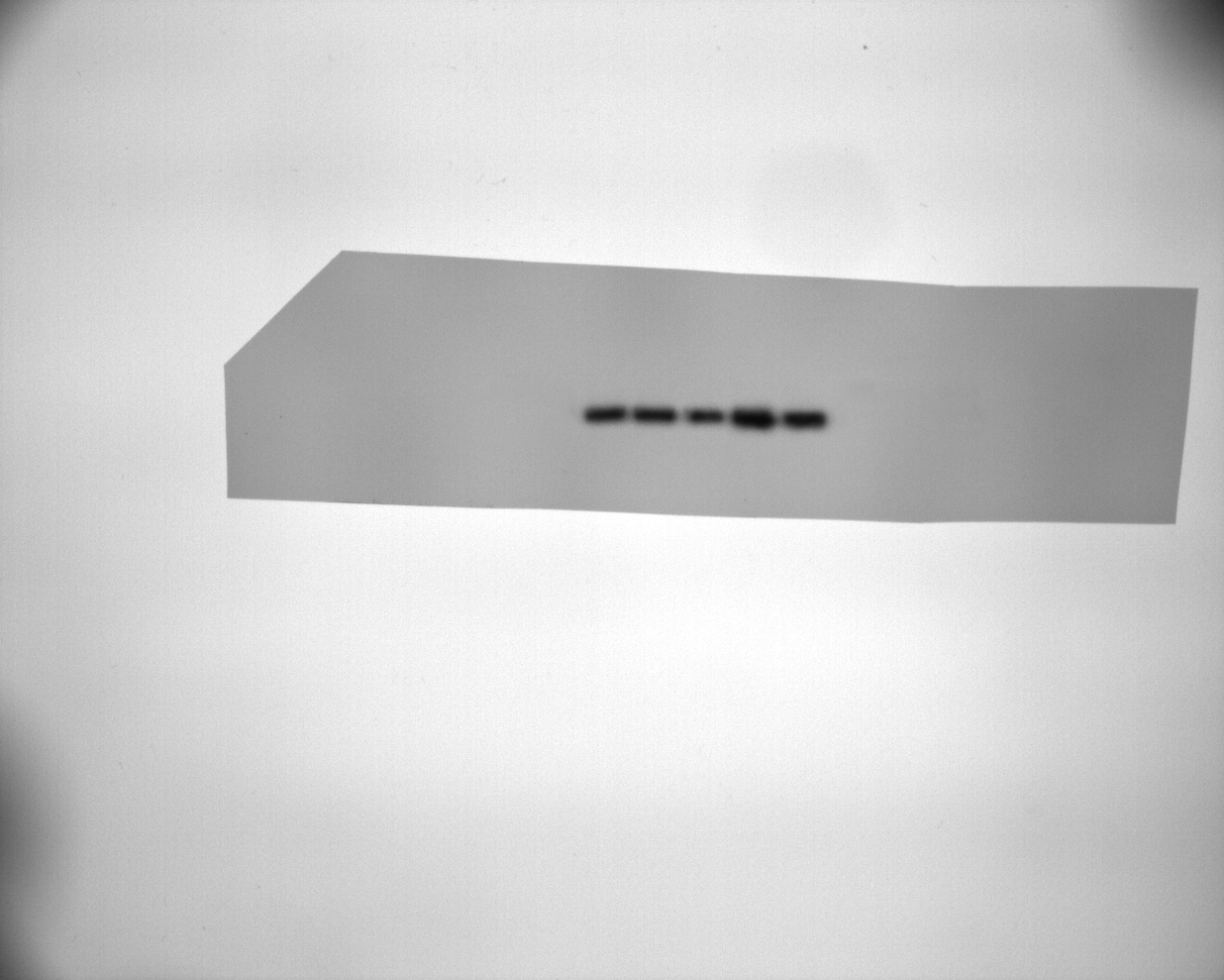


Figure 3D _GAPDH


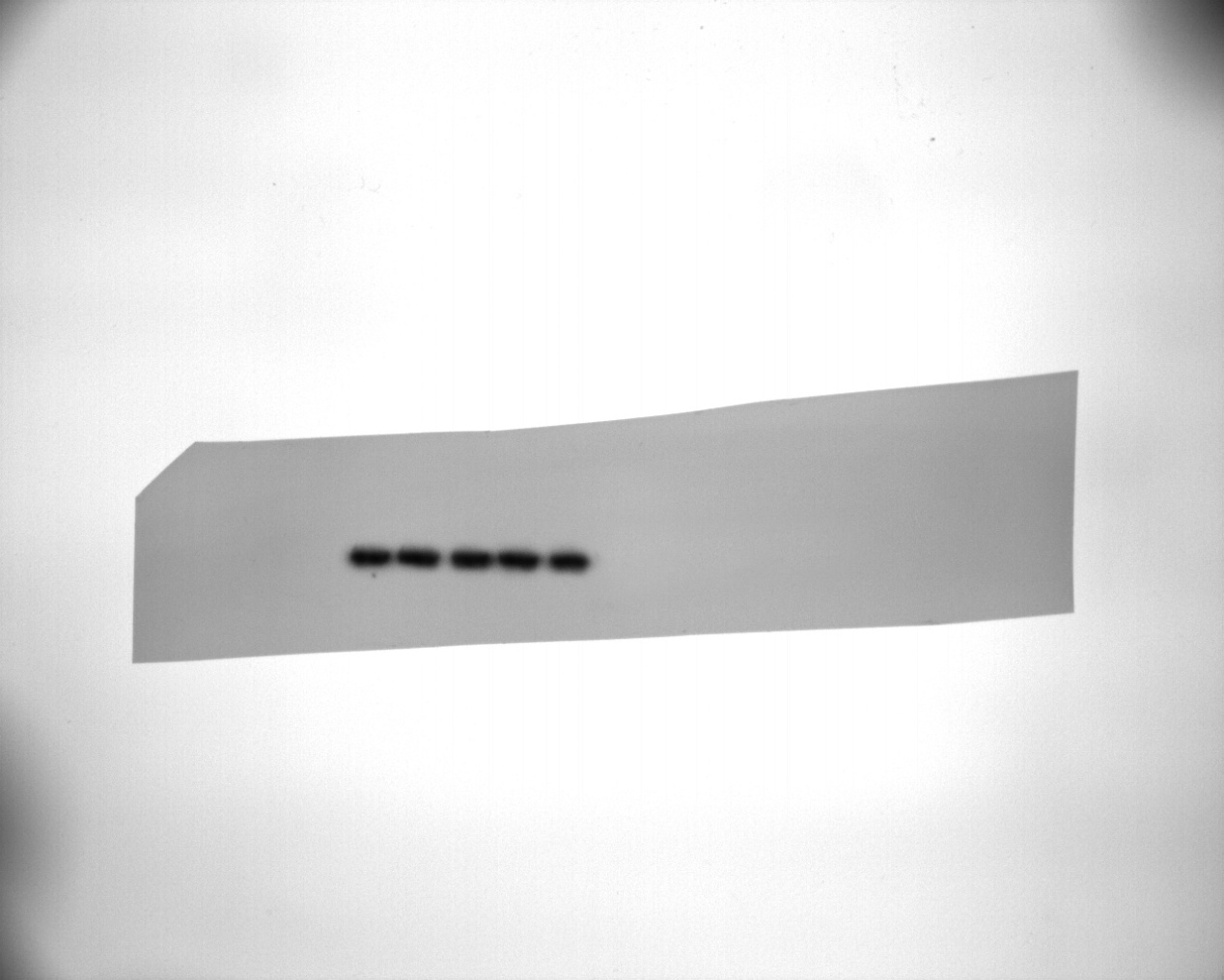


Figure 5D _α-SMA


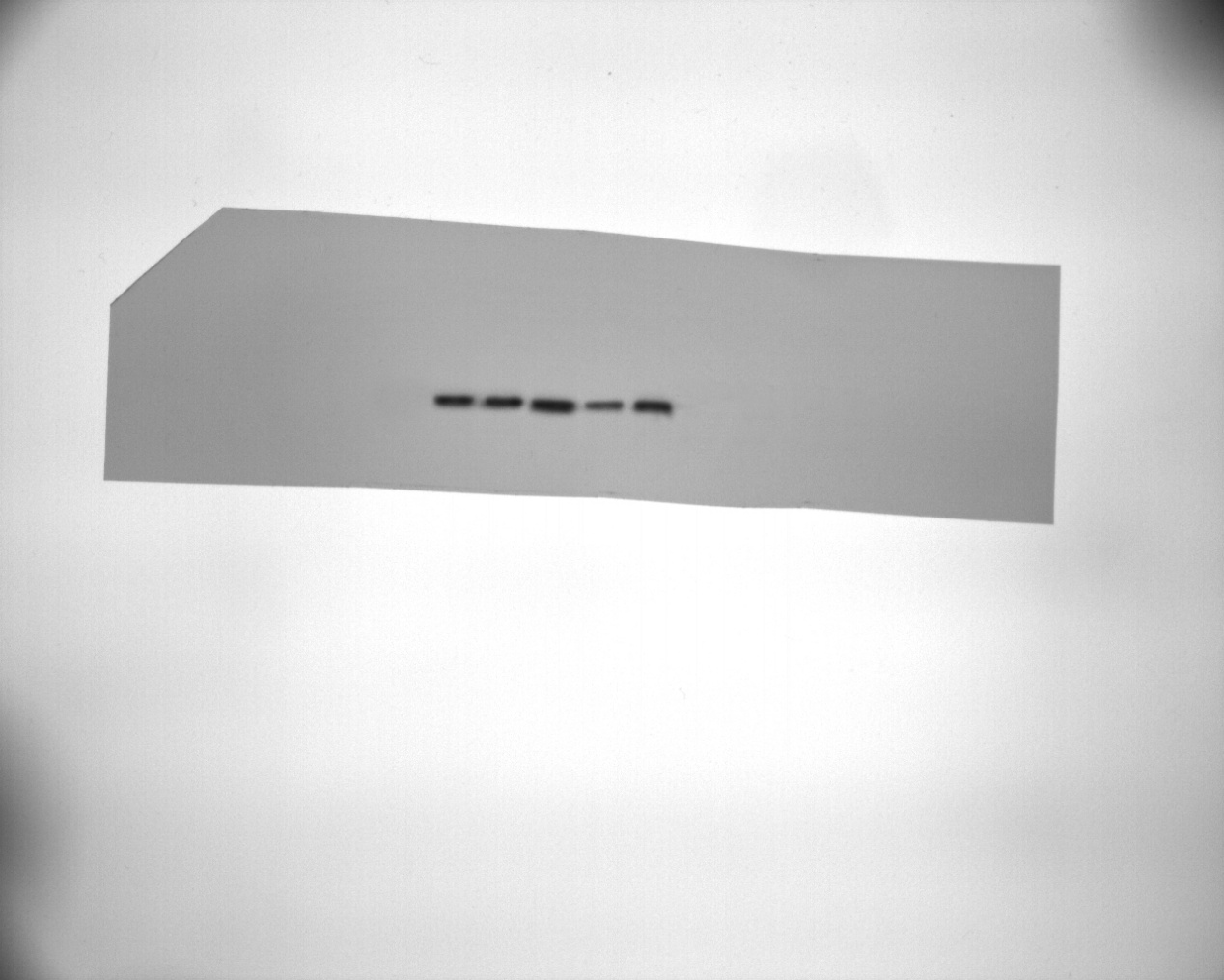


Figure 5D _OPN


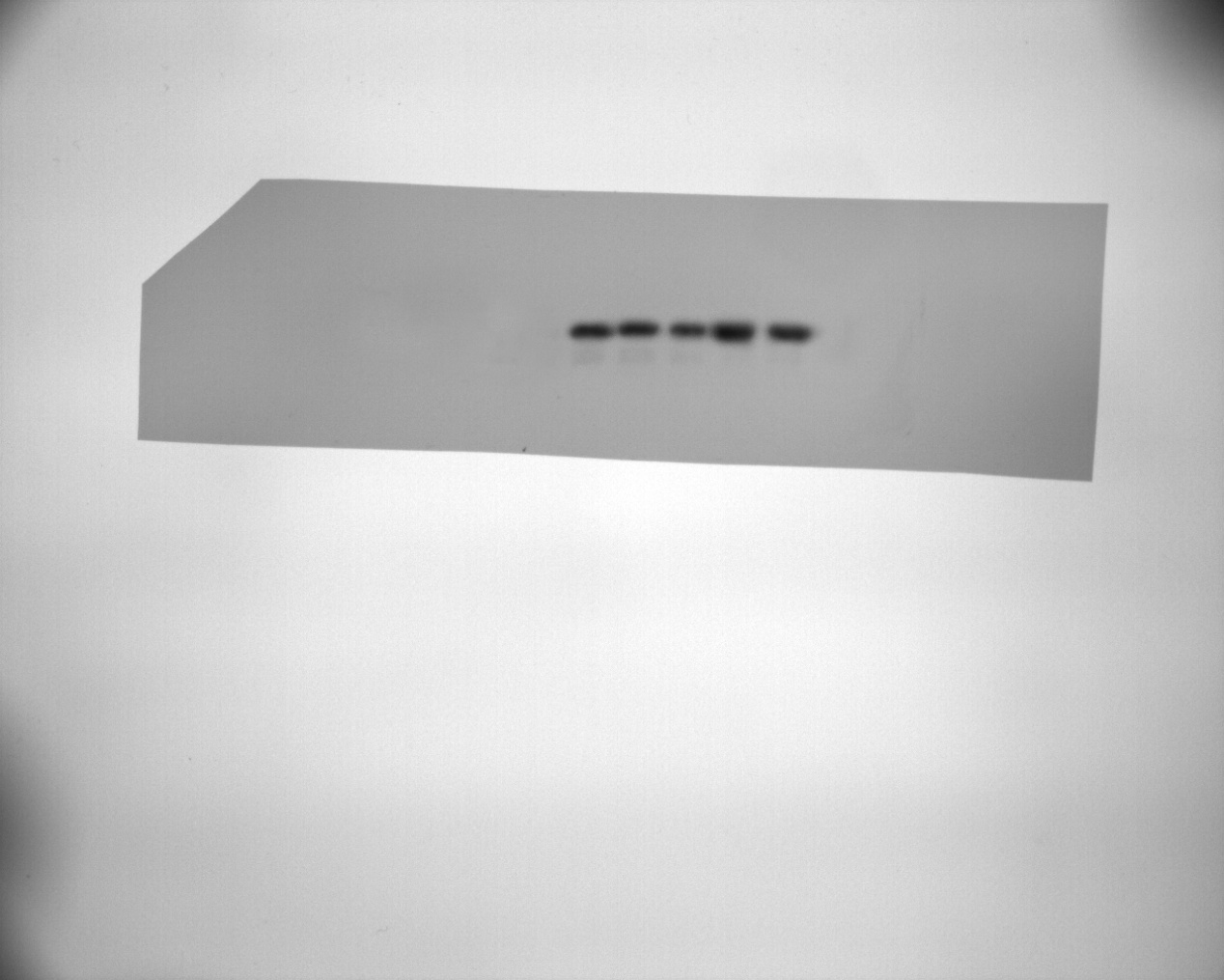


Figure 5D _MMP2


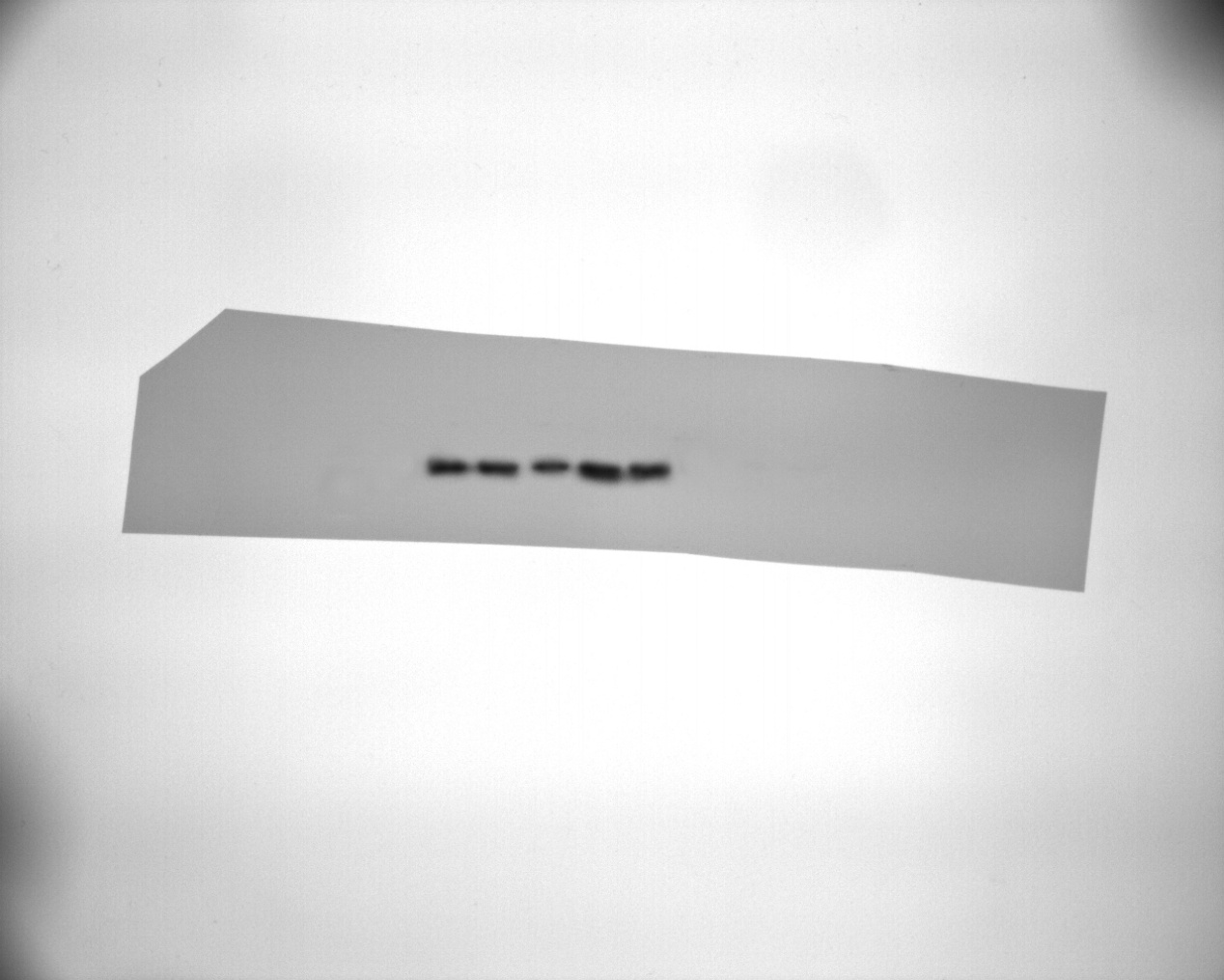


Figure 5D _MMP9


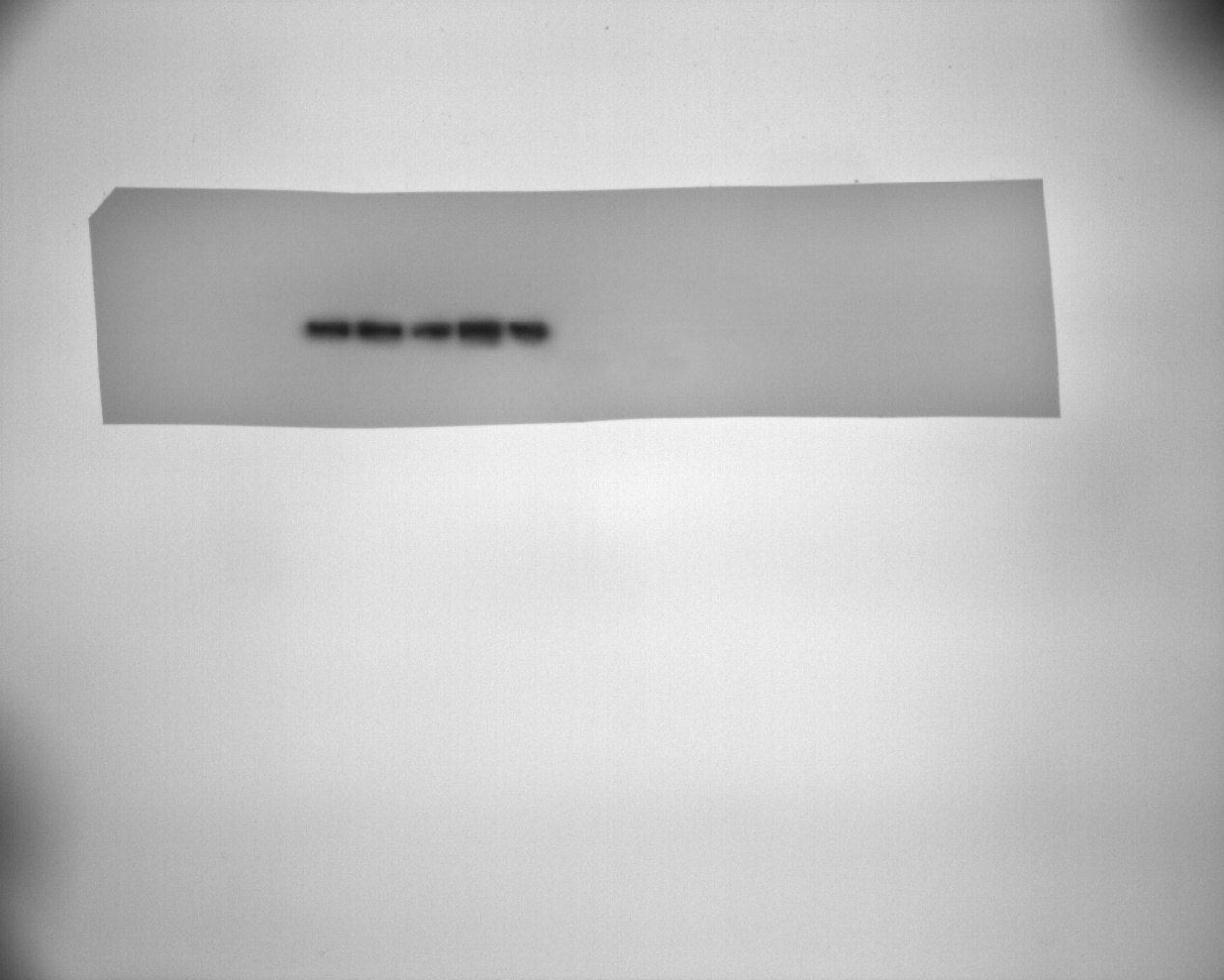


Figure 5D _GAPDH


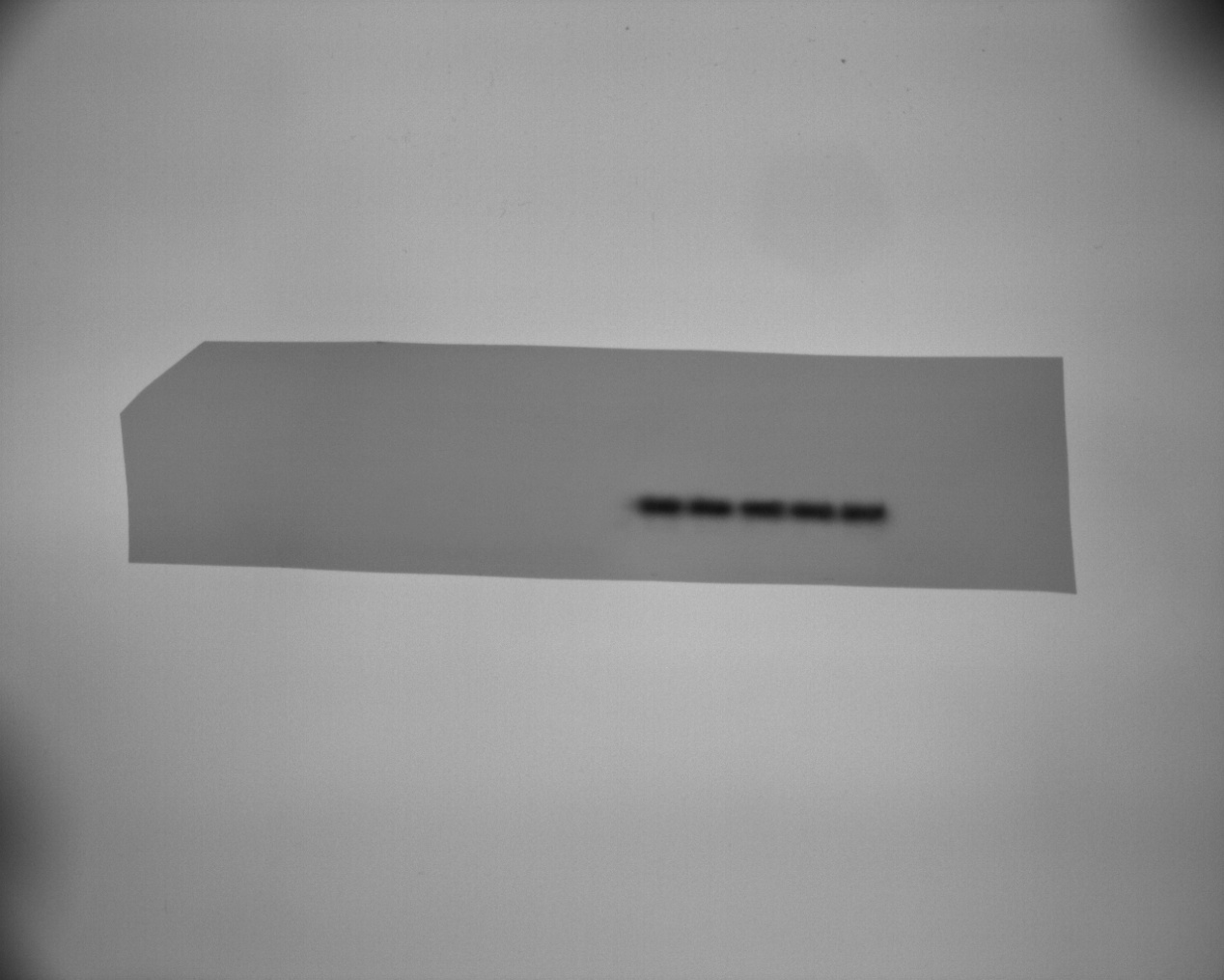


Figure 6A _smad2


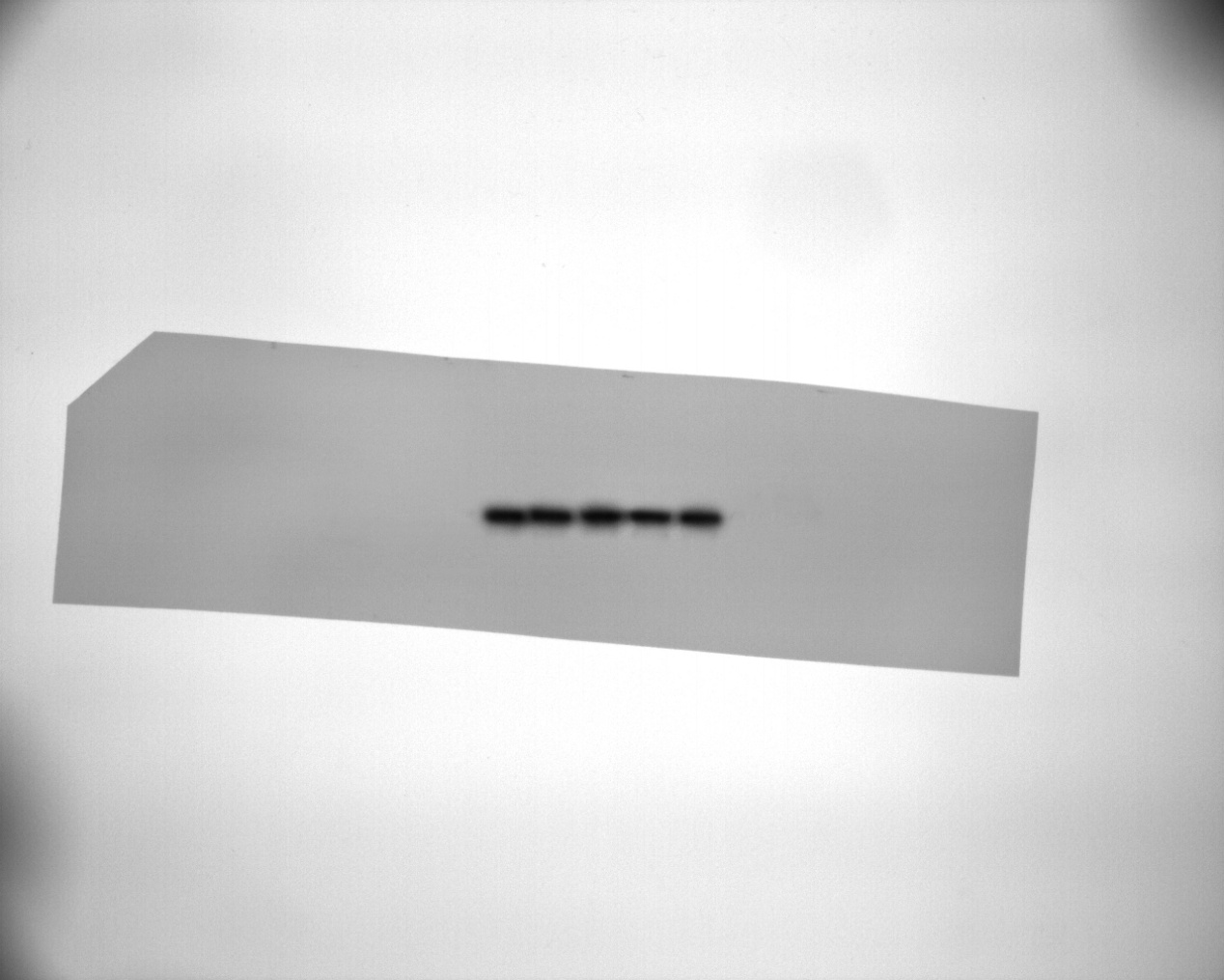


Figure 6A _p-smad2


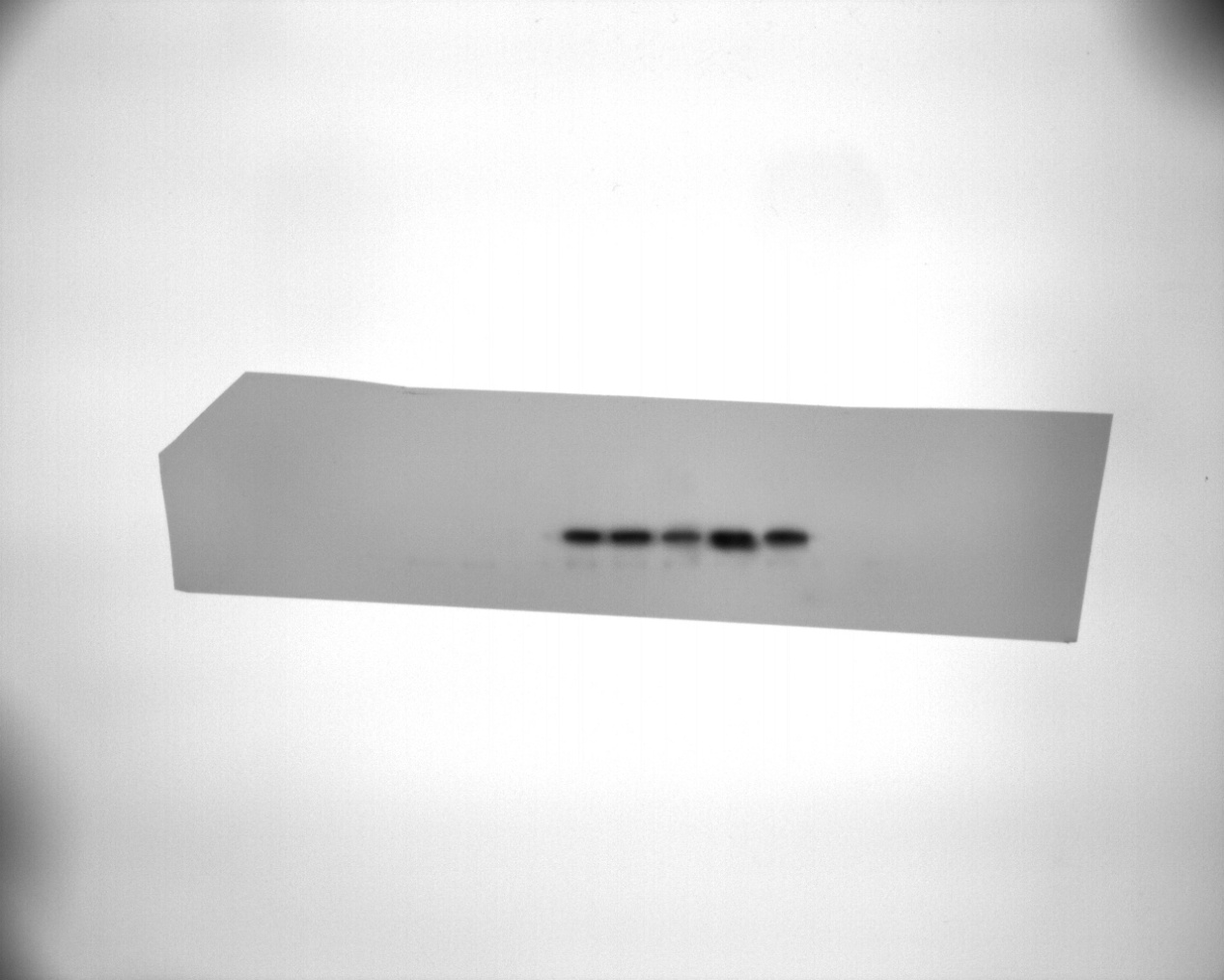


Figure 6A _smad3


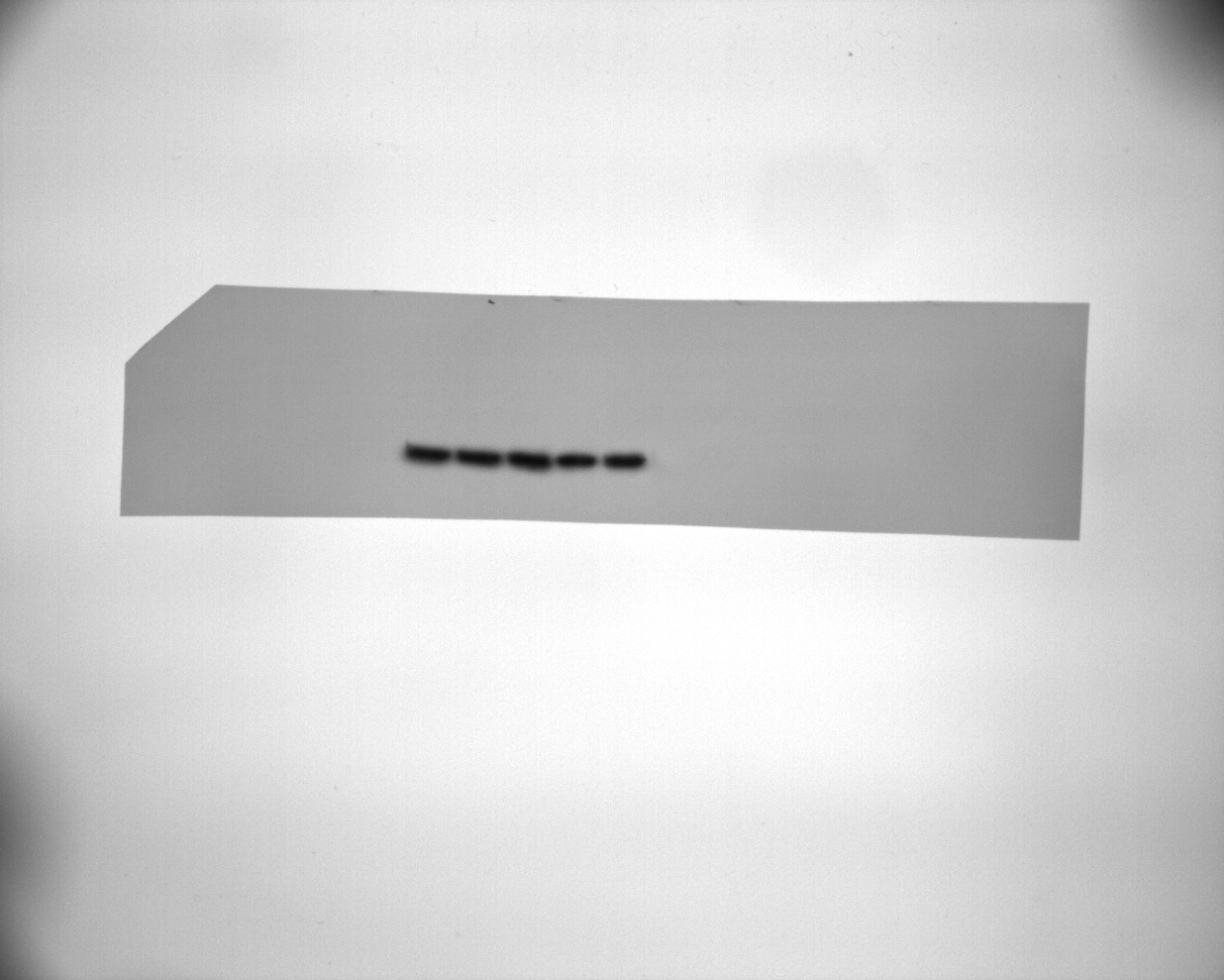


Figure 6A _p-smad3


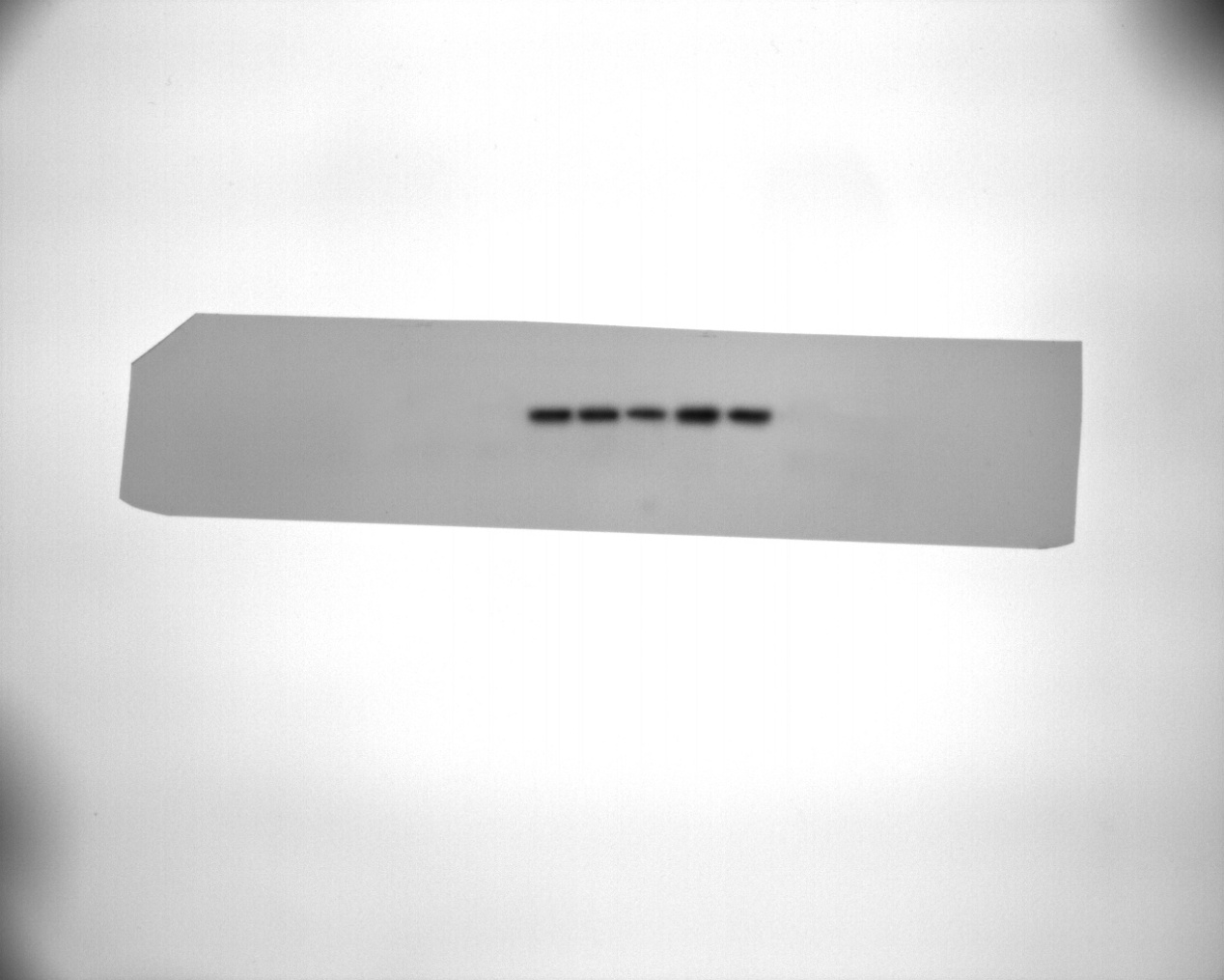


Figure 6A _GAPDH


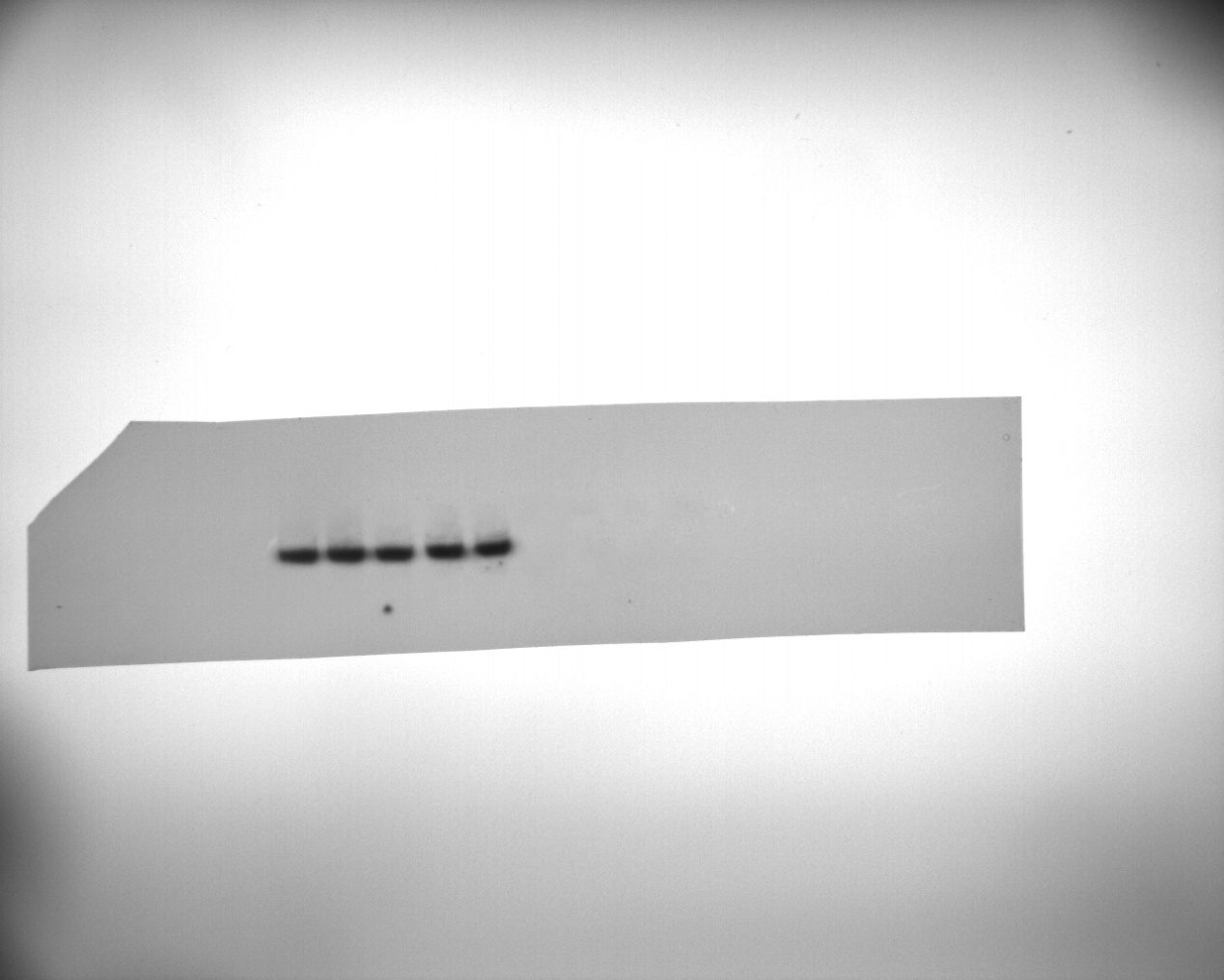

Supplement: Supplementary file 3 [file Table_2.docx]
