# Supplementary figures and images for: Silencing MALAT1 represses pathological progression, inflammation, and vascular smooth muscle cell phenotype switching by regulating the SEMA3C-mediated Smad pathway in intracranial aneurysms
Source: Front Cell Neurosci. 2026 Mar 11;20:1706518. doi: 10.3389/fncel.2026.1706518 (PMC13013063; doi:10.3389/fncel.2026.1706518)

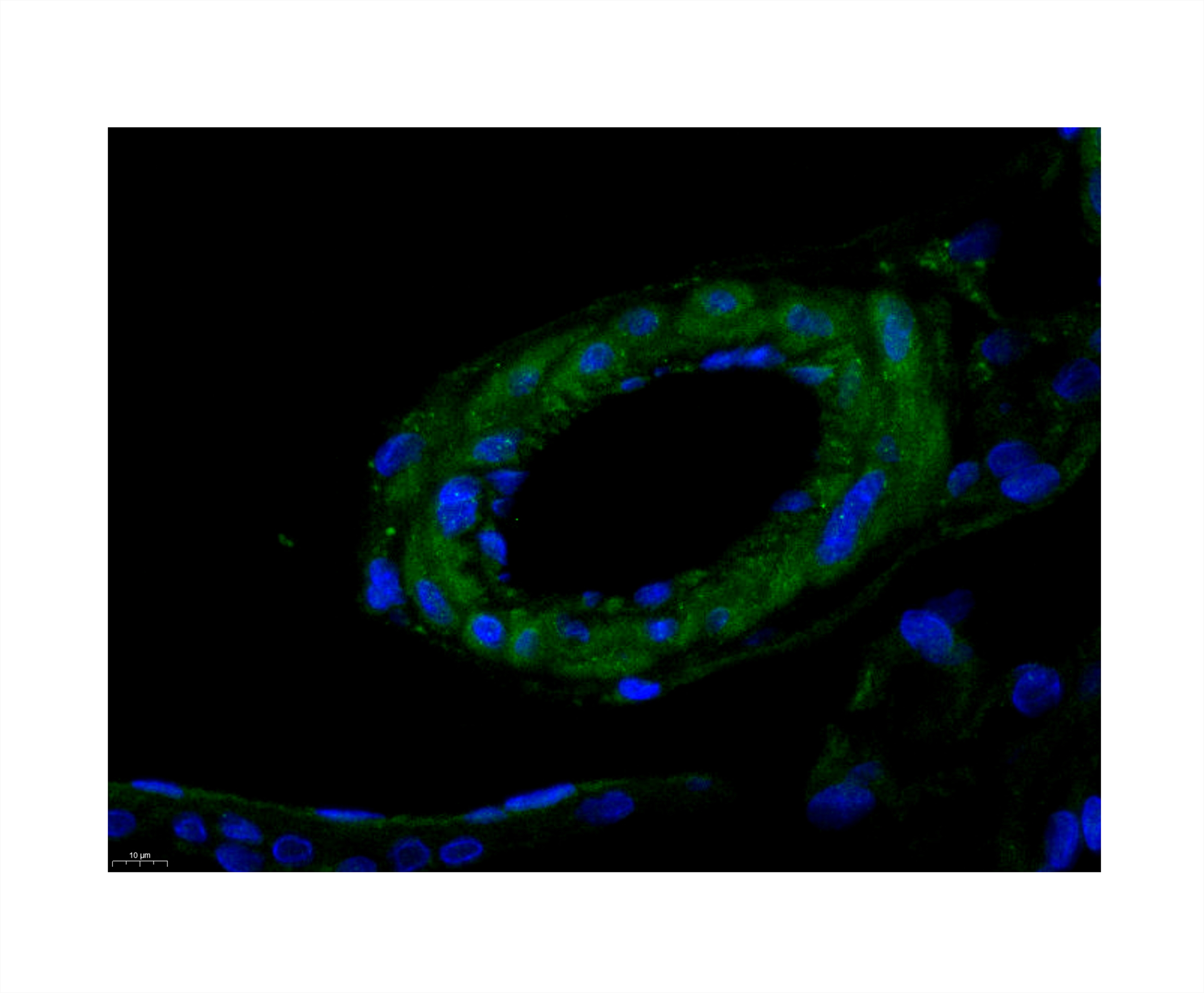

Supplement: Supplementary Figure 1 — Observation of the infected efficiency of lentivirus by fluorescent marker gene method. [file Image_1.tif]

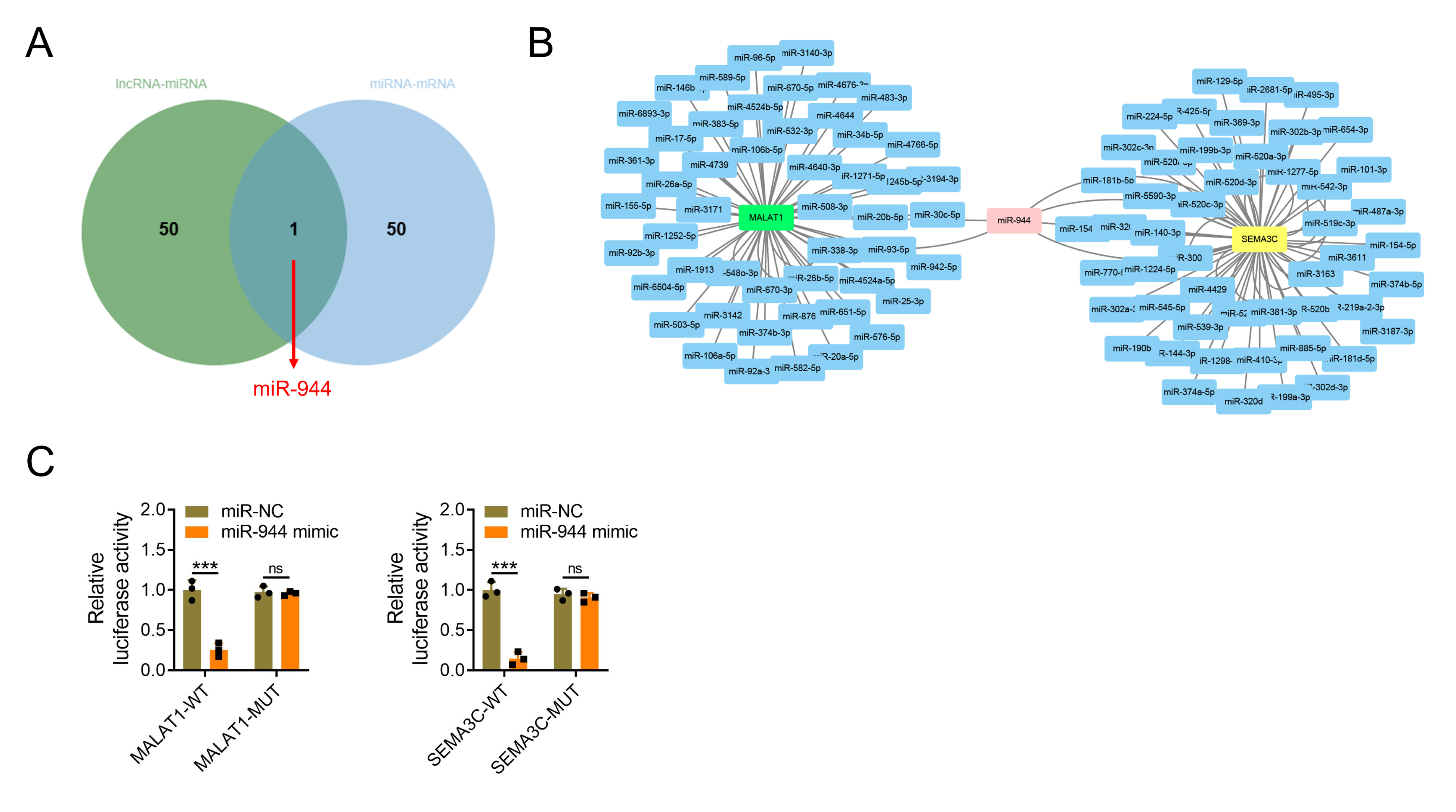

Supplement: Supplementary Figure 2 — Relation between MALAT1 and SEMA3C. Venn diagram between the top 50 predicted miRNAs targeting MALAT1 and the top predicted miRNAs targeting SEMA3C (A), ceRNA network between MALAT1 and SEMA3C (B), and luciferase reporter gene experiments (C). [file Image_2.tif]

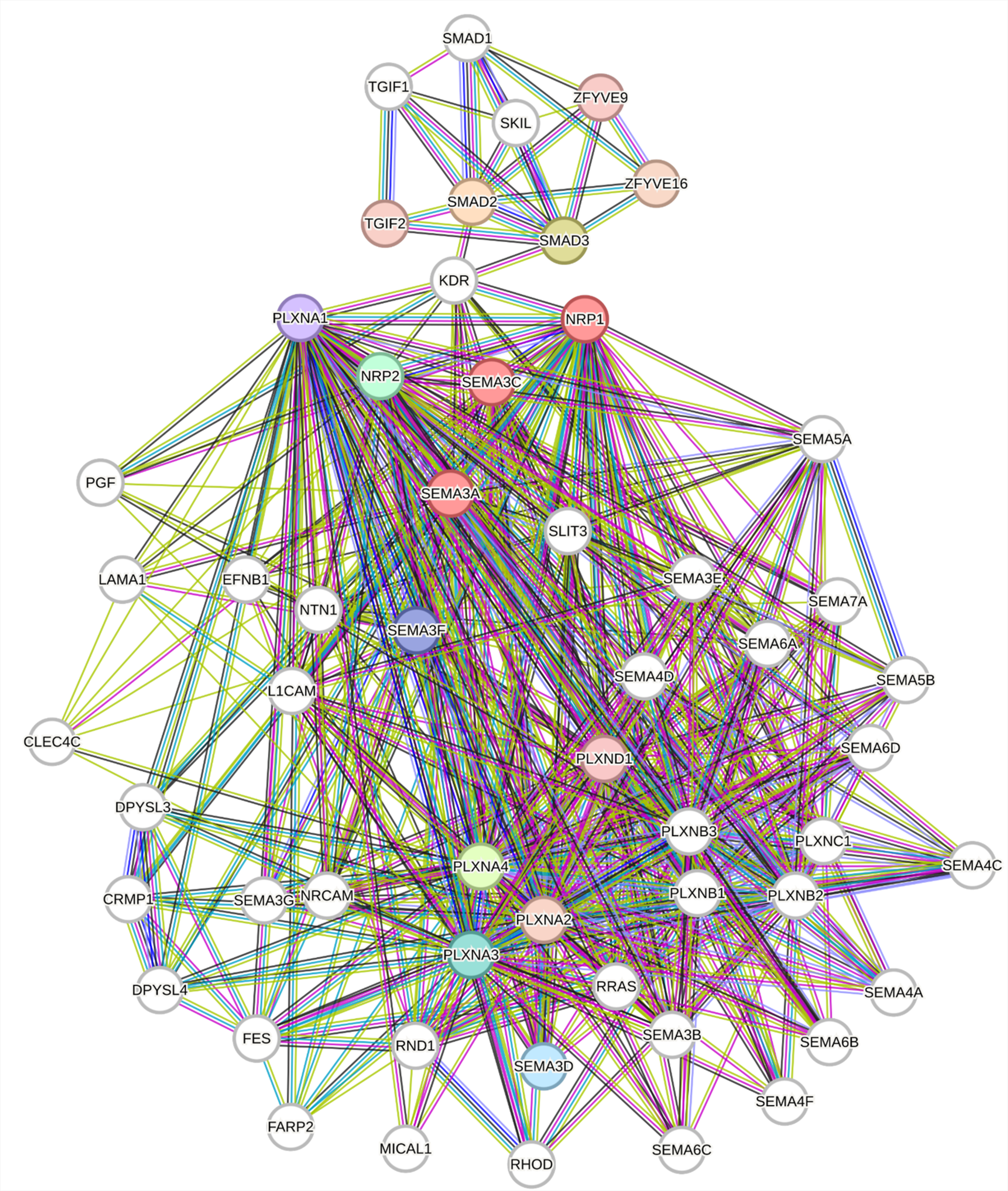

Supplement: Supplementary Figure 3 — Protein-protein interaction analysis. [file Image_3.tif]
